# Inhibition of pneumococcal growth and biofilm formation by human isolates of *Streptococcus mitis* and *Streptococcus oralis*

**DOI:** 10.1128/aem.01336-24

**Published:** 2025-02-26

**Authors:** João Borralho, Sara Handem, João Lança, Bárbara Ferreira, Catarina Candeias, Adriano O. Henriques, N. Luisa Hiller, Carina Valente, Raquel Sá-Leão

**Affiliations:** 1 Laboratory of Molecular Microbiology of Human Pathogens, Instituto de Tecnologia Química e Biológica António Xavier, Universidade Nova de Lisboa 98819 , Oeiras, Portugal; 2 Laboratory of Microbial Development, Instituto de Tecnologia Química e Biológica António Xavier 98819 , Oeiras, Portugal; 3 Department of Biological Sciences, Carnegie Mellon University 6612 , Pittsburgh, Pennsylvania, USA; 4 Polytechnic Institute of Castelo Branco 70919 , Castelo Branco, Portugal; Washington University in St. Louis, St. Louis, Missouri, USA

**Keywords:** *Streptococcus pneumoniae*, *Streptococcus mitis*, *Streptococcus oralis*, biotherapeutic, bacteriocin, colonization, biofilm

## Abstract

In a world facing the unprecedented threat of antibiotic-resistant bacteria, targeted approaches to control colonization and prevent disease caused by common pathobionts offer a promising solution. *Streptococcus pneumoniae* (pneumococcus) is a leading cause of infections worldwide, affecting both children and adults despite available antimicrobials and vaccines. Colonization, which occurs in the form of a biofilm in the upper respiratory tract, is frequent and a prerequisite for disease and transmission. The use of live bacterial strains as biotherapeutics for infectious diseases is actively being explored. Here, we investigated the potential of commensal streptococci to control *S. pneumoniae*. Screening of over 300 human isolates led to the identification of seven strains (one *Streptococcus oralis* and six *Streptococcus mitis*, designated A22 to G22) with inhibitory activity against *S. pneumoniae* of multiple serotypes and genotypes. Characterization of A22 to G22 cell-free supernatants indicated the involvement of secreted proteins or peptides in the inhibitory effect of all *S. mitis* isolates. Genome analyses revealed the presence of 64 bacteriocin loci, encoding 70 putative bacteriocins, several of which are novel and absent or rare in over 7,000 publicly available pneumococcal genomes. Deletion mutants indicated that bacteriocins partially or completely explained the anti-pneumococcal activity of the commensal strains. Importantly, strains A22 to G22 were further able to prevent and disrupt pneumococcal biofilms, a proxy for nasopharyngeal colonization. These results highlight the intricacy of the interactions among nasopharyngeal colonizers and support the potential of strains A22 to G22 to be used as live biotherapeutics, alone or in combination, to control *S. pneumoniae* colonization.

**IMPORTANCE:**

*Streptococcus pneumoniae* (pneumococcus) infections remain a major public health issue despite the use of vaccines and antibiotics. Pneumococci asymptomatically colonize the human upper respiratory tract, a niche shared with several commensal *Streptococcus* species. Competition for space and nutrients among species sharing the same niche is well documented and tends to be more intense among closely related species. Based on this rationale, a screening of several commensal streptococci isolated from the human upper respiratory tract led to the identification of strains of *Streptococcus mitis* and *Streptococcus oralis* capable of inhibiting most pneumococcal strains, across diverse serotypes and genotypes. This inhibition was partially or wholly linked to the expression of novel bacteriocins. The selected *S. mitis* and *S. oralis* strains significantly disrupted pneumococcal biofilms, indicating a potential for using commensals as biotherapeutics to control pneumococcal colonization, a key step in preventing disease and transmission.

## INTRODUCTION

*Streptococcus pneumoniae* (pneumococcus) is a leading cause of morbidity and mortality worldwide, affecting both children and adults despite the availability of antimicrobial therapies and vaccines. Annually, approximately 300,000 children under 5 years old die from pneumococcal infections, and nearly 3.7 million suffer from severe infections (1). The burden among adults is significantly higher but remains poorly defined (2). While multivalent pneumococcal conjugate vaccines are highly effective, they target only a subset of the over 100 described serotypes, leading to serotype replacement over time (3–7). The emergence of multidrug-resistant *S. pneumoniae* strains compromises treatment options (8). Furthermore, antibiotics have long-term effects by disturbing the microbiota and imposing global selective pressure for resistance (9). These challenges have spurred interest in developing alternative and complementary approaches to treat or prevent pneumococcal infections (9, 10).

The World Health Organization (WHO) recommends that new interventions aiming to decrease pneumococcal disease should target colonization (11). Pneumococcal colonization, which occurs in the form of a biofilm in the upper respiratory tract (URT), is frequent and a prerequisite for disease and transmission (12). Two recent studies have described strategies aimed at targeting pneumococcal colonization. One proposed the use of human endogenous bile salts (13); the other resorted to phage-derived endolysins active against *S. pneumoniae* (14). Yet, another strategy could be the use of bacterial strains with anti-pneumococcal activity.

The use of bacterial strains with probiotic traits for targeted therapeutic approaches is being actively explored by both academic groups and biotechnological companies to control bacterial infections (15, 16). Examples include (i) engineered probiotic *Escherichia coli* strains able to reduce vancomycin-resistant enterococci and *Pseudomonas aeruginosa* in the gut of animal models (17, 18); (ii) a phase 1 randomized clinical trial, where a *Staphylococcus hominis* strain reduced the bacterial load of *Staphylococcus aureus* in the skin of participants with atopic dermatitis (19); (iii) the use of *Streptococcus salivarius* and *Streptococcus oralis* to prevent pharyngotonsillitis in children (20, 21); and (iv) the use of *Streptococcus dentisani* to improve oral health (22). Notably, several studies have shown the protective effect of gut bacteria against respiratory tract infections (RTIs) (reviewed in reference [23]). For example, a randomized trial with over 4,000 newborns has shown reduced incidence of RTIs and sepsis when administered with a combination of *Lactobacillus plantarum* and fructo-oligosaccharide (24). Nonetheless, to the best of our knowledge, no studies have addressed the use of bacteria to specifically target pneumococcal colonization.

It is well established that in the polymicrobial environment of the URT, commensal bacteria and pathobionts compete for space and nutrients. In particular, *S. pneumoniae* shares the niche with several other *Streptococcus* spp., commonly regarded as true commensals (25, 26). Isolates of such closely related species likely compete for the same resources and as such they could possibly be used to control *S. pneumoniae* colonization.

While the mechanisms by which biotherapeutics act are diverse, bacteriocins are often implicated. Bacteriocins are proteins or peptides produced by bacteria for bacterial warfare (27). They typically have a narrow activity spectrum, targeting bacteria closely related to the producer (27). They have been identified as a potential alternative to conventional antimicrobials to fight multi-resistant bacteria (27, 28). Successful examples include the use of thuricin CD, produced by *Bacillus thuringiensis*, to treat *Clostridioides difficile* infections (29); and the identification of lugdunin, a novel peptide produced by *Staphylococcus lugdunensis* that inhibits nasal colonization by *S. aureus* (30). Streptococcal species encode several classes of bacteriocins and, given the genomic plasticity of these species, within each class, the molecules are highly diverse (27).

The aim of this study was to identify commensal streptococcal strains with the ability to inhibit pneumococci and, thus, with the potential to be used as biotherapeutics against pneumococcal colonization. Screening of over 300 isolates led to the identification of seven strains (one *S*. *oralis* and six *Streptococcus mitis*) with inhibitory activity against a diverse collection of *S. pneumoniae*. The cell-free supernatant of these seven strains also displayed inhibitory activity, which, in the case of *S. mitis* strains, was lost upon protease treatment, suggesting the involvement of secreted proteins or peptides in the observed inhibition. Genome analysis of these commensals revealed the presence of a total of 64 bacteriocin loci, encoding 70 putative bacteriocins, most of which were rare or absent in a collection of over 7,000 publicly available pneumococcal genomes. Deletion mutants of these loci indicated that the anti-pneumococcal activity of the commensal strains could be partially or completely explained by bacteriocins. Importantly, the commensal strains effectively inhibited *S. pneumoniae* in dual-species biofilms, with the combined use of all seven strains being the most effective.

## RESULTS

### Identification of seven commensal *S. oralis* or *S. mitis* streptococci strains with broad anti-pneumococcal activity

**Fig 1:**
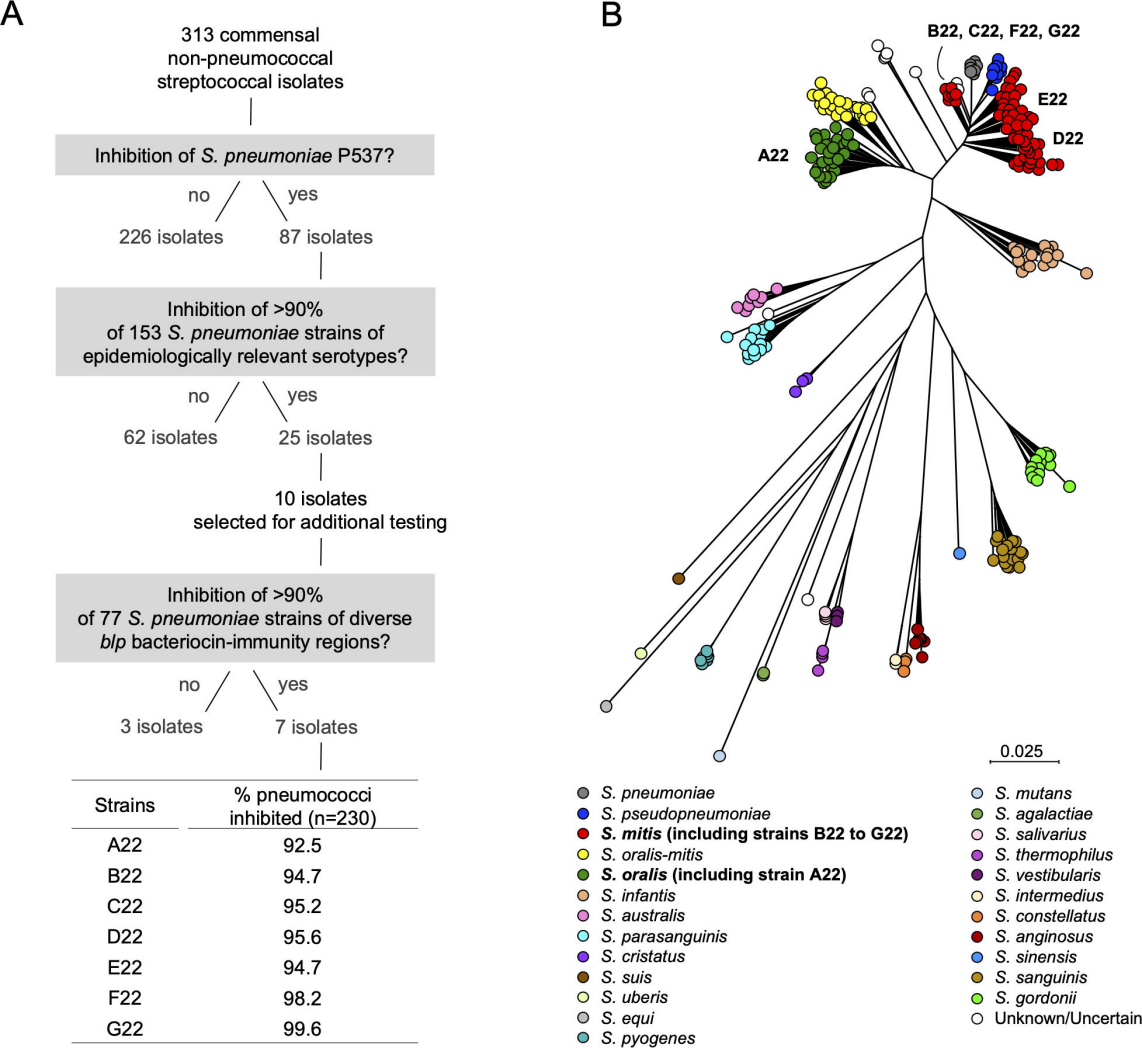
Identification of *S. oralis* and *S. mitis* isolates with broad anti-pneumococcal activity. (**A**) A collection of 313 non-pneumococcal streptococcal isolates was screened for anti-pneumococcal activity against the *blp*-bacteriocin susceptible *S. pneumoniae* strain P537 (34) through overlay assays. Isolates inhibiting P537 were further screened for inhibitory activity against 153 pneumococci representing epidemiologically relevant serotypes. Seven isolates, named strains A22 to G22, inhibited at least 90% of that *S. pneumoniae* collection and were next tested against additional *S. pneumoniae* strains (*n* = 77) with diverse BIRs in the *blp* locus. Combining the inhibition results of the two pneumococcal collections, strains A22 to G22 inhibited 92.5%–99.5% of the 230 pneumococci. (**B**) Species assignment of strains A22 to G22 was done by constructing a neighbor-joining phylogenetic tree using concatenated sequences of seven housekeeping genes described for the viridans multilocus sequence analysis scheme (36). The Jukes-Cantor model was used for nucleotide distance measure, and bootstrap analysis was performed based on 500 replicates. Scale bar reflects the number of nucleotide substitutions per site.

To identify commensal streptococci with the ability to inhibit *S. pneumoniae*, we selected strains previously isolated as non-pneumococcal streptococci (*n* = 313) (7, 31–33). These strains were then tested by overlay assays for inhibitory activity against *S. pneumoniae*. These strains were isolated from nasopharyngeal swabs of healthy children and adults who were not colonized with *S. pneumoniae* at the time of sampling and with no antimicrobial consumption in the month preceding sampling (7, 31–33). These strains were tested against an array of phylogenetically diverse pneumococcal strains (*n* = 230), encoding 30 distinct serotypes (including 17 targeted by the 20-valent pneumococcal conjugate vaccine and 16 by the 21-valent pneumococcal conjugate vaccine) and 157 multilocus sequence typing (MLST) types all isolated from nasopharyngeal and oropharyngeal samples (Table S1). First, the 313 isolates were initially tested against the *S. pneumoniae* strain P537, a serotype 6A strain with the deletion of the *blp* bacteriocin locus, which makes it susceptible to many bacteriocins and a good reporter of inhibitory activity (34). Eighty-seven isolates prevented the growth of P537, and these were further tested against a diverse set of 153 *S*. *pneumoniae* strains. The screen yielded seven isolates, hereinafter named strains A22 to G22, which inhibited at least 90% of that *S. pneumoniae* collection. Finally, these were tested against an additional 77 *S*. *pneumoniae* strains with diverse bacteriocin-immunity regions (BIRs) in the *blp* locus (35). In total, each of the A22 to G22 inhibited >90% of the total *S. pneumoniae* strains (Fig. 1A; Table S1).

The genomes of strains A22 to G22 were sequenced, assembled, and annotated (Table S2). To determine the species of strains A22 to G22, a multilocus sequence analysis (MLSA) scheme for viridans group streptococci (based on internal sequences of seven housekeeping genes) was used (36). The resulting neighbor-joining phylogenetic tree indicated that strain A22 clustered with the *S. oralis* cluster, whereas strains B22 to G22 clustered with *S. mitis* (Fig. 1B). Thus, we have identified seven commensal streptococci strains, one *S*. *oralis* and six *S*. *mitis*, with broad inhibitory activity against a library of epidemiologically relevant pneumococcal strains.

### The pneumococcal inhibitory activity of strains A22 to G22 is attributed to secreted gene products

**Fig 2:**
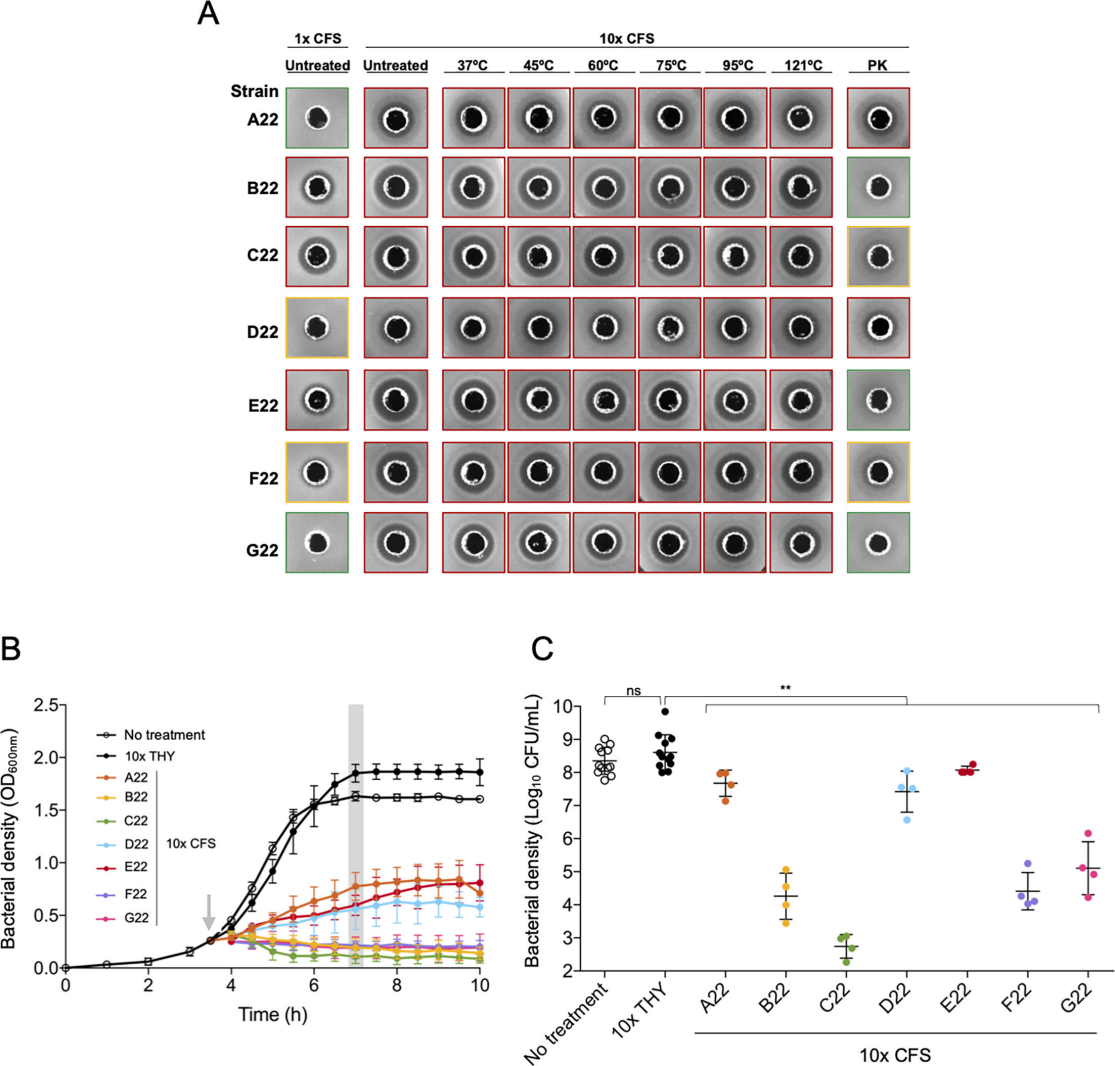
Supernatants of strains A22 to G22 inhibit *S. pneumoniae*. (**A**) Cell-free supernatants of strains A22 to G22 were obtained from the late exponential phase (OD_600nm_ of 1.0) THY-grown cultures and 10-fold concentrated. CFSs were heat treated (by incubation at 37°C, 45°C, 60°C, and 75°C for 1.5 h, at 95°C for 1 h, and at 121°C for 20 min) and protease treated (incubation with 1 mg/mL proteinase K [PK] at 37°C for 3 h) and tested for inhibitory activity against the indicator *S. pneumoniae* strain P537 through well-diffusion assays. Inhibition halos above 11 mm (red outline) indicate inhibitory activity; inhibition halos of 9–10 mm (yellow outline) indicate residual inhibitory activity; and inhibition halos of 8 mm (well width, green outline) indicate no inhibitory activity. (**B**) Early exponential phase cultures (OD_600nm_ of 0.3) of *S. pneumoniae* P537 were treated with 10× CFS, 10× THY, or remained untreated (as indicated by the gray arrow), and OD_600nm_ was monitored every 30 min. Gray bar indicates the time point at which an aliquot was taken for cell viability counts. Mean and standard deviation of at least four biological replicates are shown for each time point. (**C**) Cell viability of P537 was assessed 3 h post-treatment with 10× CFS. Geometric mean and geometric standard deviation of at least four biological replicates are represented for each condition. **P-value < 0.01, Mann-Whitney *U* test with Benjamini and Hochberg correction for FDR; statistical comparison was done with treatment with 10× THY; ns, not significant.

To test whether the inhibitory activity displayed by strains A22 to G22 against *S. pneumoniae* was due to secreted molecules, as anticipated for bacteriocins, cell-free supernatants (CFSs) were obtained in early-stationary phase, concentrated 10-fold (10× CFS), and tested for inhibitory activity against *S. pneumoniae* P537 strain in well-diffusion assays. The non-concentrated CFS of five of the seven strains (B22 to F22) had inhibitory activity. The 10× CFS of all seven strains had inhibitory activity, which was greater than the one observed in the non-concentrated CFS, with strains B22, C22, E22, F22, and G22 displaying the strongest effect (Fig. 2A). The inhibitory activity of 10× CFS was not affected by heat treatment, up to 121°C. In contrast, protease treatment either abolished or greatly reduced the activity of most CFS, the exception being supernatants from strains A22 and D22 (Fig. 2A).

To evaluate if CFS of strains A22 to G22 affected planktonic growth, a culture of *S. pneumoniae* strain P537 in the early-exponential phase was treated with the 10× CFS. All CFS significantly impaired the growth of P537, with the CFS of strains B22, C22, F22, and G22 showing the strongest impact (Fig. 2B). These results were extended by measuring the cell viability of the pneumococcal strain P537, which showed a decrease in viability 3 h post-treatment (Fig. 2C).

Taken together, these results indicate that strains A22 to G22 release thermostable molecules, most likely of proteinaceous nature (with the exception of strains A22 and D22), that inhibit *S. pneumoniae* growth on solid and liquid medium.

### Strains A22 to G22 carry an arsenal of genes encoding putative bacteriocins and immunity proteins

To understand if some of the inhibitory activity of strains A22 to G22 was due to the production of bacteriocins, we conducted an *in silico* search for bacteriocins in the annotated genomes of the seven strains. A diverse repertoire of bacteriocin-like gene clusters was found and characterized for content and organization. We identified four types of loci, namely bacteriocin-like peptide (*blp*), competence-associated bacteriocin (*cab*), streptococcins (lactococcin 972-like), and lantibiotics (37–40). The genome of *S. oralis* strain A22 encoded three bacteriocin-related loci, whereas each of the genomes of *S. mitis* strains (B22 to G22) encoded 9–11 bacteriocin-related loci (Table 1; Fig. S1). The search revealed many paralogs to previously characterized systems. It also revealed two novel streptococcin (lactococcin 972-like) loci, named streptococcin F (*scf*) and G (*scg*), as well as two novel lantibiotic loci in *S. mitis*, which we named mitilancidin (*mld*) and mitilacticin (*mlc*) (Fig. S2). Interestingly, in all *S. mitis* strains, we also captured several incomplete bacteriocin-related loci, which lacked bacteriocin genes but encoded other genes, including immunity proteins (Fig. S2). We propose these loci play a defensive role against exogenous bacteriocins.

**TABLE 1:** The genomes of strains A22 to G22 encode several bacteriocin-related loci^*a*^

Strain	Bacteriocin loci				Total no. of loci
	Blp	Competence	Streptococcin (lactococcin 972-like)	Lantibiotic	
A22	*blp2*	*cab6*	*scc*		3
B22	*blp1, iblp5.1*	*cab1, icib*	*isca2, iscb2, iscc, isce2*	*slk*	9
C22	*blp1, blp4, blp6*	*cab4, icib*	*sce, isca2, iscc, iscq*	***mlc*** *, slk*	11
D22	*blp3*	*cab5, cab8*	*scb, isca1, iscc*	*ipld, isbo, inis*	9
E22	*blp1, blp4*	*cab2, cib*	*isca3, iscc, isce1*	***mld**, slk, ilan*	10
F22	*blp1, iblp5.2, blp6*	*cab3, cib*	***scf*** *, isca1, iscb1, isce4*		9
G22	*blp1, blp4*	*cab7, icib*	***scg*** *, isca2, iscb2, iscc, isce3*	*slk*	10

^*a*^ Four types of bacteriocin-related gene loci were identified based on genetic content and organization: bacteriocin-like peptide (*blp*), competence-associated bacteriocin (*cab*), streptococcin (lactococcin 972-like), and lantibiotic. Loci lacking bacteriocin genes (but otherwise containing genes characteristic of bacteriocin loci) were considered incomplete and are indicated with an *“i”* as prefix. Loci underlined indicate those for which deletion mutants of bacteriocin-containing regions were constructed. Loci highlighted in bold are first described in this study.

In total, we identified genes encoding 70 distinct putative bacteriocins and 119 distinct putative immunity proteins. To establish the distribution of these genes across pneumococcal strains, we used a reference collection of 7,548 genomes of *S. pneumoniae* (41). Notably, 80% of the bacteriocin- and 92% of the immunity protein-encoding genes were absent from the reference collection (41) when using strict criteria of 100% query length coverage and 100% amino acid sequence identity. When using more relaxed criteria of ≥90% sequence coverage and ≥80% of identity, we found that 80% of the putative bacteriocins and 50% of the immunity proteins described in this study were absent or rare among *S. pneumoniae* strains (Table S3). Additionally, we investigated the distribution of these genes across a collection of 322 *S*. *mitis* and 108 *S*. *oralis* genomes. When using the stricter criteria, 89% and 92% of bacteriocin-encoding genes were found to be absent among the *S. mitis* and *S. oralis* genomes, respectively, or around 55% for both species when using the relaxed criteria. In addition, 77% and 94% of immunity-encoding genes were absent among the *S. mitis* and *S. oralis* genomes, respectively, using the stricter criteria, and 11% and 41% when using the relaxed criteria.

Collectively, these analyses revealed that strains A22 to G22 contain an extensive array of bacteriocin-related genes that could explain their broad inhibitory activity against *S. pneumoniae*.

### Multiple regulatory elements are encoded upstream of the putative bacteriocin loci of strains A22 to G22 and blp-like loci respond to quorum-sensing peptides

**Fig 3:**
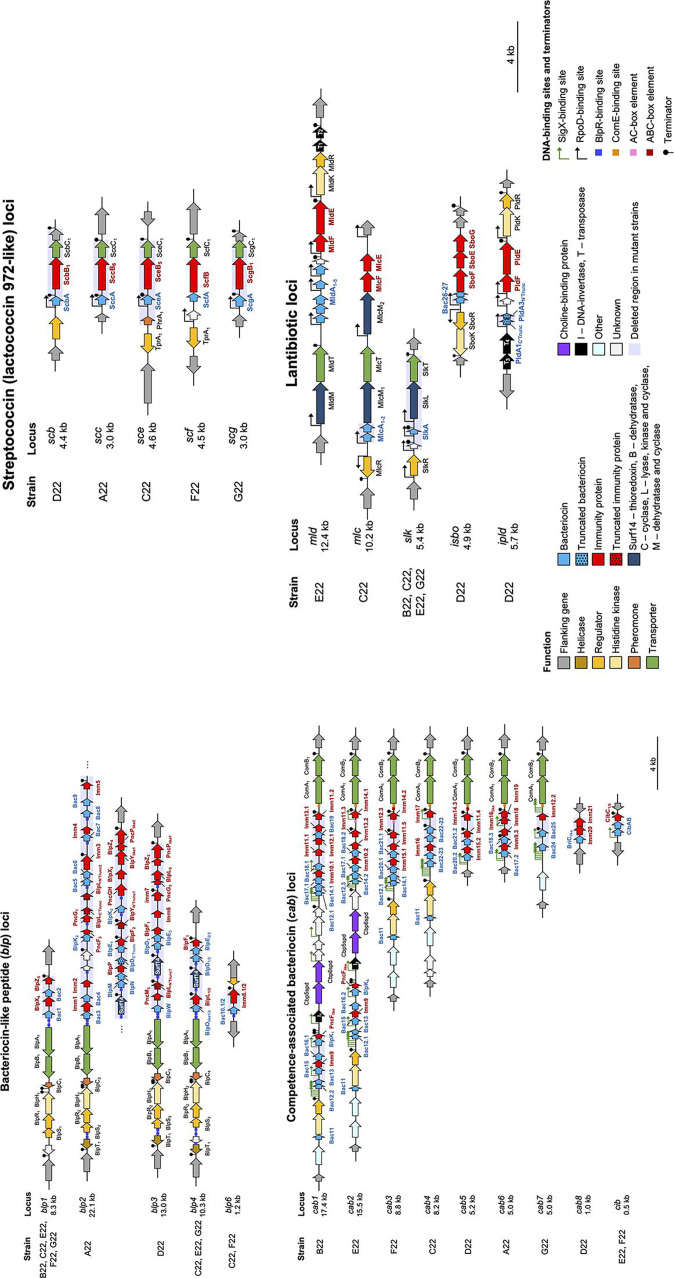
The genomes of strains A22 to G22 encode multiple bacteriocin loci. A schematic representation of gene clusters is shown. Genes are colored by function, and flanking genes are represented in gray. Names of putative bacteriocins and immunity proteins are indicated in blue and red, respectively. Other relevant proteins are indicated in black. Numbers after gene names indicate allelic variants. Locus size (excluding the flanking genes) is indicated next to the locus name. Putative DNA-binding sites (SigX, RpoD, BlpR, ComE, and AC-, ABC-box elements) and terminator regions are also represented. Deletion-mutant strains were constructed for bacteriocin-containing regions, indicated by light purple boxes. This was done for all strains with the exception of strain E22 and locus *scf* from strain F22. Locus tags of the represented regions (genes from left to right) are the following: *blp1*—SMIB22_19330–19210, SMIC22_19450–19330, SMIE22_04050–04170, SMIF22_19410–19290, SMIG22_20130–2010; *blp2*—SORA22_03770–04130; *blp3*—SMID22_17960–17760; *blp4*—SMIC22_15990–15830, SMIE22_15420-15250, SMIG22_16660–16500; *blp6*—SMIC22_12570–12540, SMIF22_12710–12680; *cab1*—SMIB22_00640–00940; *cab2*—SMIE22_00500-00750; *cab3*—SMIF22_00400–00560; *cab4*—SMIC22_00520–00670; *cab5*—SMID22_00350–00420; *cab6*—SORA22_00820–00900; *cab7*—SMIG22_00540–00590; *cab8*—SMID22_18840–18800; *cib*—SMIE22_18370-18400, SMIF22_18970–19000; *scb*—SMID22_02310–02360; *scc*—SORA22_14540–14500; *sce*—SMIC22_05670–05730; *scf*—SMIF22_03120–03070; *scg*—SMIG22_15150–15110; *mld*— SMIE22_18640-18490; *ipld*—SMID22_05170–05260; *mlc*—SMIC22_19190–19110; *slk*—SMIB22_16070–16020, SMIC22_16240–16190, SMIE22_15680-15620, SMIG22_16890–16840; *isbo*—SMID22_12430–12340.

To gain insight into the regulation of the putative bacteriocin gene clusters, we first conducted an *in silico* analysis of their regulatory regions (promoters and terminators) (Tables S4 and S5; Fig. 3; Fig. S3). We found consensus binding sequences for the housekeeping sigma factor RpoD (also named σ70) (42) in the promoter regions of the lantibiotic and streptococcin (lactococcin 972-like) loci. For *blp* and *cab* loci, the promoter regions had consensus binding sequences for transcriptional regulators associated with quorum-sensing systems (Fig. S4). Specifically, we identified ComE (early-competence transcriptional factor) and SigX (late-competence sigma factor) binding sequences in the *cab* loci (43) and BlpR-binding sequences in the blp locus (43, 44). Additionally, as previously shown for *S. pneumoniae* (45), strains D22, E22, and F22 had BOX elements upstream of the competence transporter *comAB* operon. Of note, previous work has reported that the *cab* loci in *S. mitis* is expressed in competence consistent with the ComE and SigX boxes (42).

**Fig 4:**
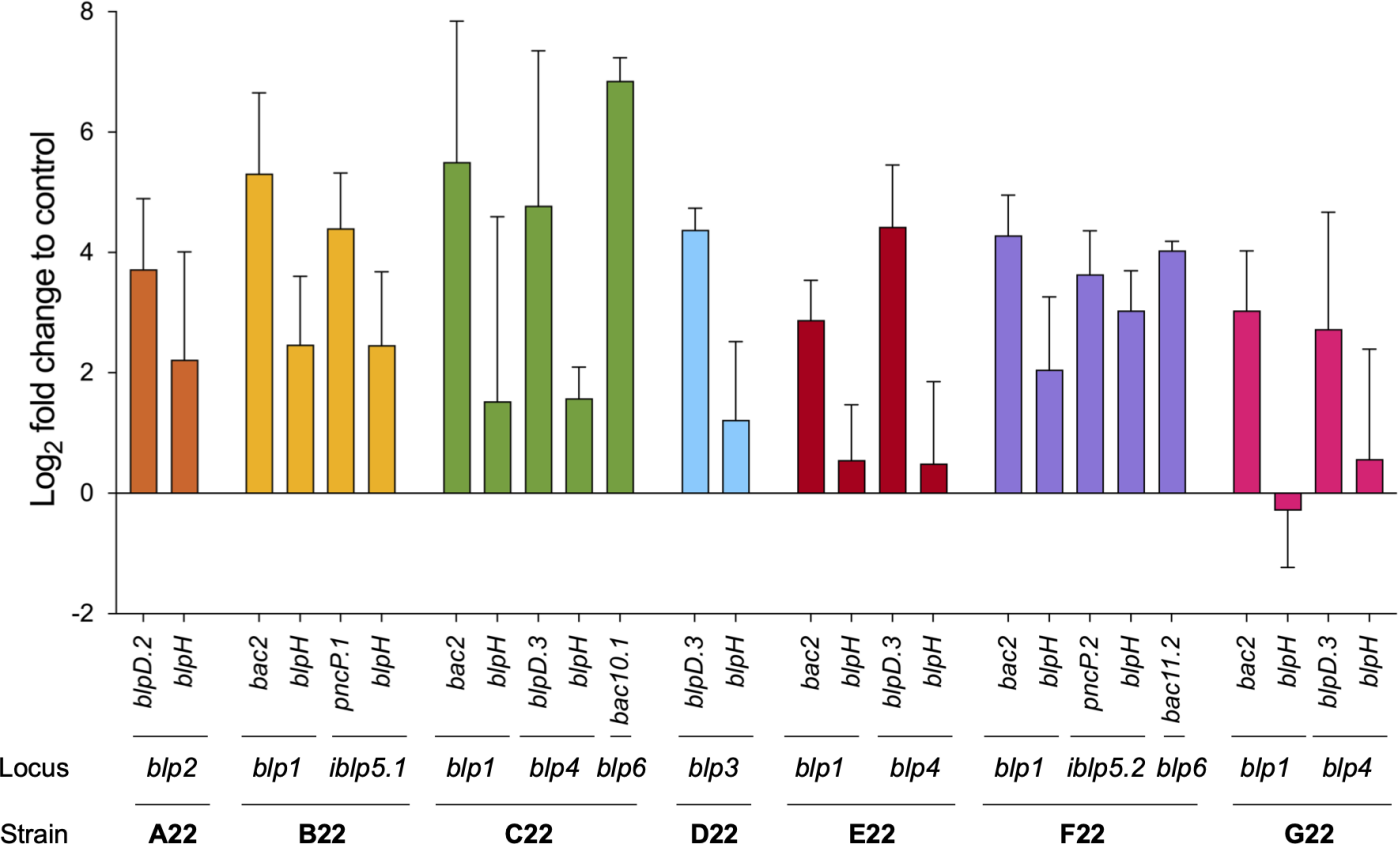
The bacteriocin-like peptide loci of strains A22 to G22 are expressed and controlled by the BlpC pheromone. Gene expression was measured by qRT-PCR after exposure of strains for 5 min to synthetic cognate BlpC. Loci *iblp5.1*, *iblp5.2*, and *blp6* lack a cognate BlpC; hence, their gene stimulation was done with the addition of *blp1* BlpC of the same strain. The *Y* axis shows fold change differences after BlpC treatment compared to the untreated culture. Error bars represent the standard deviation of the mean of three biological replicates.

Characterized streptococcal *blp* loci are driven by secreted autoinducing BlpC peptides that activate the BlpH/R two-component histidine kinase signaling system (46). To investigate whether the regulation of *blp* loci in strains A22 to G22 was quorum-sensing dependent, we tested their response to BlpC. We generated synthetic peptides matching the product of the *blpC* gene encoded in their genomes (Table S6) and used qRT-PCR to measure the expression of the regulatory gene *blpH* and one gene from the bacteriocin and immunity region (Fig. 4). For *iblp5.1*, *iblp5.2,* and *blp6*, which lack *blpC* (and thus the corresponding cognate pheromone), stimulation was achieved with BlpC, coded by *blp1* (present in the same strains) (Fig. 1). Addition of BlpC increased transcript levels of most genes when compared to the untreated control (except for some *blpH*), suggesting that the *blp* loci of strains A22 to G22 are expressed and regulated by quorum sensing (Fig. 4). Moreover, the increased expression of genes of *iblp5.1*, *iblp5.2,* and *blp6* loci indicated cross-regulation between various *blp* loci occurring in the same strain (Fig. 4).

We conclude that the bacteriocin-related loci encoded by the genomes of strains A22 to G22 are expressed in laboratory conditions and thus, could at least in part, contribute to the inhibitory activity of strains A22 to G22. Additionally, for loci encoding blp-bacteriocins, expression was quorum-sensing dependent in the sense that it was stimulated by exogenous synthetic pheromones.

### Bacteriocin loci of *S. mitis* strains are implicated in pneumococcal inhibition

To evaluate the individual contribution of the putative bacteriocin loci to *S. pneumoniae* inhibition, we constructed deletion mutants lacking each bacteriocin-immunity region independently. We were able to produce mutants for all but one bacteriocin-related locus containing putative bacteriocins that were identified *in silico* in six of the seven strains (the exception being locus *scf* in strain F22, and strain E22 which was not transformable in any of the conditions tested). A total of 28 mutants were constructed (Table 1; Fig. 3) and tested for activity in solid and liquid media.

**Fig 5:**
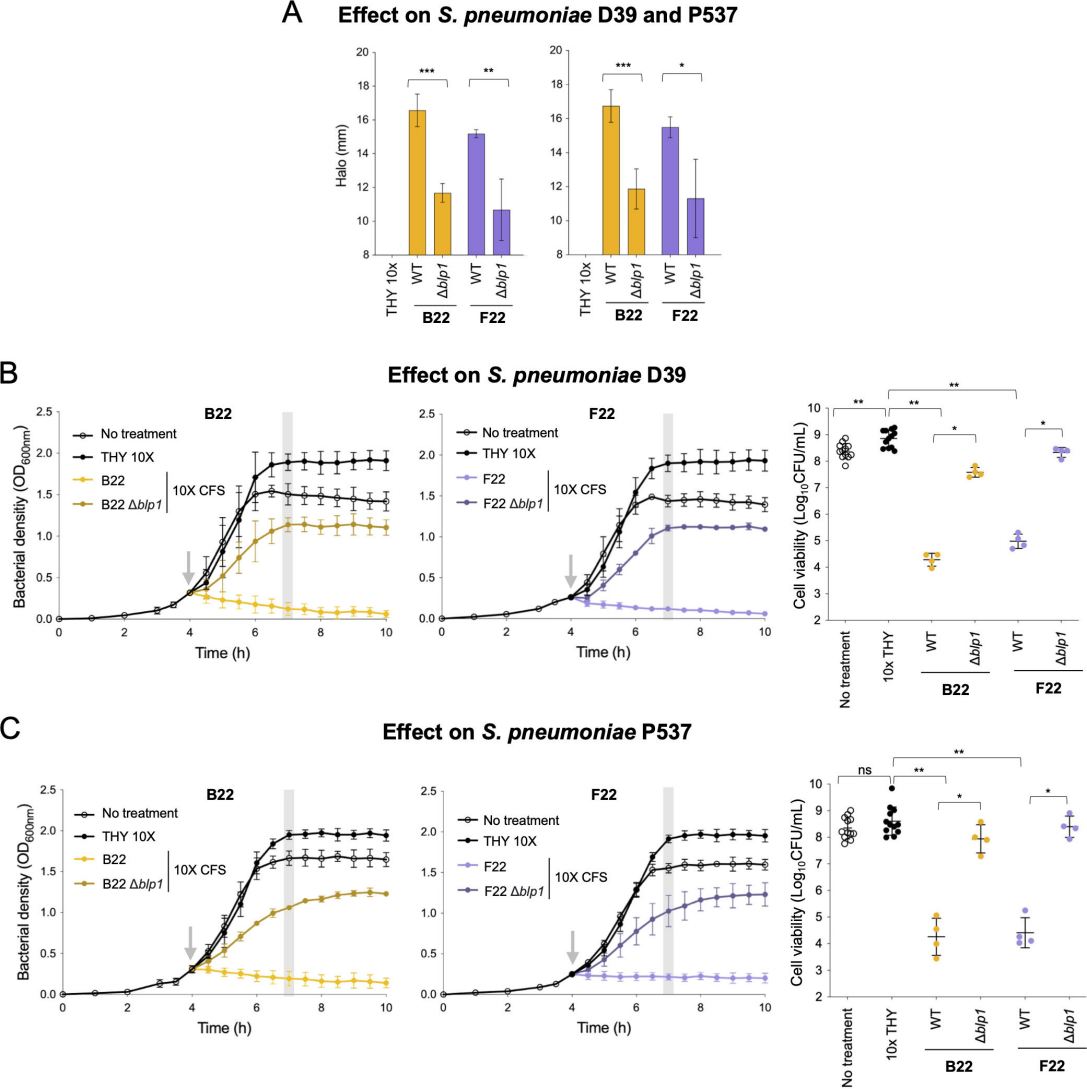
Locus *blp1* from *S. mitis* strains B22 and F22 negatively affects the growth and cell viability of *S. pneumoniae* D39 and P537 strains. The CFS of *S. mitis* strains B22 and F22 and the corresponding deletion mutants lacking the bacteriocin immunity regions of locus *blp1* were tested against *S. pneumoniae* D39 and P537. (**A**) Well-diffusion assay using 10× CFS or 10× THY (control) against *S. pneumoniae* D39 (left) and P537 (right). After overnight incubation with CFS, plates were inspected for inhibition halos. The diameter of the inhibition halos was measured independently by two researchers in four independent experiments. Mean and standard deviation are represented for each condition. The inhibition halo diameter includes the well width of 8 mm. *P-value < 0.05, **P-value < 0.01, and ***P-value < 0.001, Student’s *t*-test. (**B and C**) The effect of 10× CFS on planktonic growth of *S. pneumoniae* D39 (**B**) and P537 (**C**) was assessed. Early exponential phase cultures (OD_600nm_ of 0.3) of D39 and P537 were treated with 10× CFS, 10× THY, or remained untreated (as indicated by the gray arrow) and OD_600nm_ was monitored every 30 min (left and middle panels). Gray Bar indicates the time point in which an aliquot was taken for cell viability counts. Mean and standard deviation of at least four biological replicates are represented for each condition. Cell viability (CFU/mL) of D39 and P537 was assessed 3 h post-treatment (right panel). Geometric mean and geometric standard deviation of at least four biological replicates are represented for each condition. *P-value < 0.05 and **P-value < 0.01, Mann-Whitney *U* test with Benjamini and Hochberg correction for FDR; ns, not significant.

To test for activity, we first obtained CFS of all wild-type strains and mutants and tested them against *S. pneumoniae* D39 and P537 growing in solid media (well-diffusion assays). The strongest reduction in inhibitory activity was associated with the deletion of *blp1* in strains B22 and F22 (Fig. 5A). Deletion of other bacteriocin loci also led to a reduction in inhibitory activity, although to a smaller extent. For example, loss of inhibitory effect against *S. pneumoniae* D39 was observed upon exposure of CFS of mutants of strains C22 (lacking *blp4, blp6, cab4, sce*, or *mlc*), F22 (lacking *cib*), and G22 (lacking *blp4*, *cab7,* or *slk*) (Fig. S5).

Second, we tested the same CFS of all wild-type strains and mutants against *S. pneumoniae* D39 and P537 growing in liquid media. As in the well-diffusion assays, the CFS of mutants lacking *blp1* in strains B22 and F22 showed a significant loss of inhibitory activity of *S. pneumoniae* D39 and P537 (Fig. 5B and C). In addition, mutants of strains C22 (lacking *blp1*, *blp4*, or *sce*), D22 (lacking *blp3*), and G22 (lacking *blp1*, *blp4*, or *scg*) showed a significant decrease in their ability to inhibit *S. pneumoniae* D39 and/or P537 (Fig. S6). Variability in the degree of inhibition by strains A22 to D22, F22, and G22 and its mutants on *S. pneumoniae* D39 compared to *S. pneumoniae* P537 was observed in agreement with the fact that *S. pneumoniae* is genetically diverse, including in the content of bacteriocin and immunity protein-encoding genes (Fig. S7).

**Fig 6:**
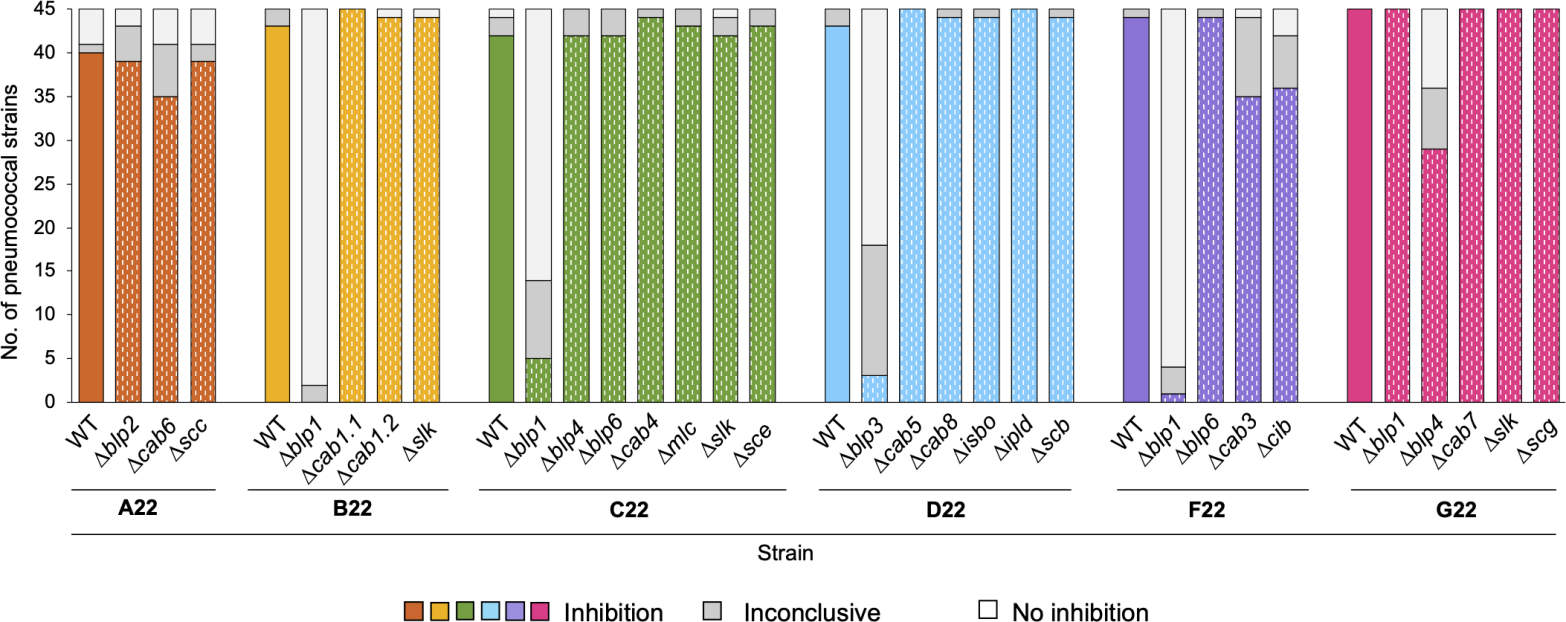
Bacteriocin-like peptide loci from *S. mitis* strains contribute to their anti-pneumococcal activity in agar overlay assays. Strains A22, B22, C22, D22, F22, and G22 and their deletion mutants (marked with dashes within the colored bars) were tested for inhibitory activity against 45 *S*. *pneumoniae* strains (representatives of 23 serotypes and 42 multilocus sequence types) in agar overlay assays. Plates were inspected for growth inhibition halos around the stabbed strains (A22, B22, C22, D22, F22, and G22 and their mutants). Results were inspected independently by two researchers, in three independent experiments.

Third, we conducted assays in solid media (agar overlay) and tested the wild-type strains and their corresponding deletion mutants against a collection of 45 *S*. *pneumoniae* isolates (representative of 23 serotypes and 42 multilocus sequence types) (Table S1). A significant loss in the capacity to inhibit most *S. pneumoniae* isolates was observed upon the deletion of *blp1* in strains B22, C22, and F22, *blp3* in strain D22, and *blp4* in strain G22 (Fig. 6).

In total, half of the mutants (14 of 28) showed reduced inhibitory activity (compared to the wild-type strain) in at least one of the assays/conditions tested (Table S8). Differences in results obtained in solid or planktonic media also suggest that bacteriocin loci may be differently expressed depending on the conditions tested.

Collectively, these results indicated that *S. mitis* strains B22, C22, D22, F22, and G22 produce bacteriocins with anti-pneumococcal activity. Furthermore, in strains B22 and F22, the bacteriocins encoded by locus *blp1* are responsible for most of the activity. Results for the *S. oralis* strain A22 supported that bacteriocin-encoding loci are not required for its activity against *S. pneumoniae*, in line with the lack of effect on activity after protease treatment of its CFS.

### The simultaneous activity of two *blp* loci of *S. mitis* strain G22 is required for full anti-pneumococcal inhibition

**Fig 7:**
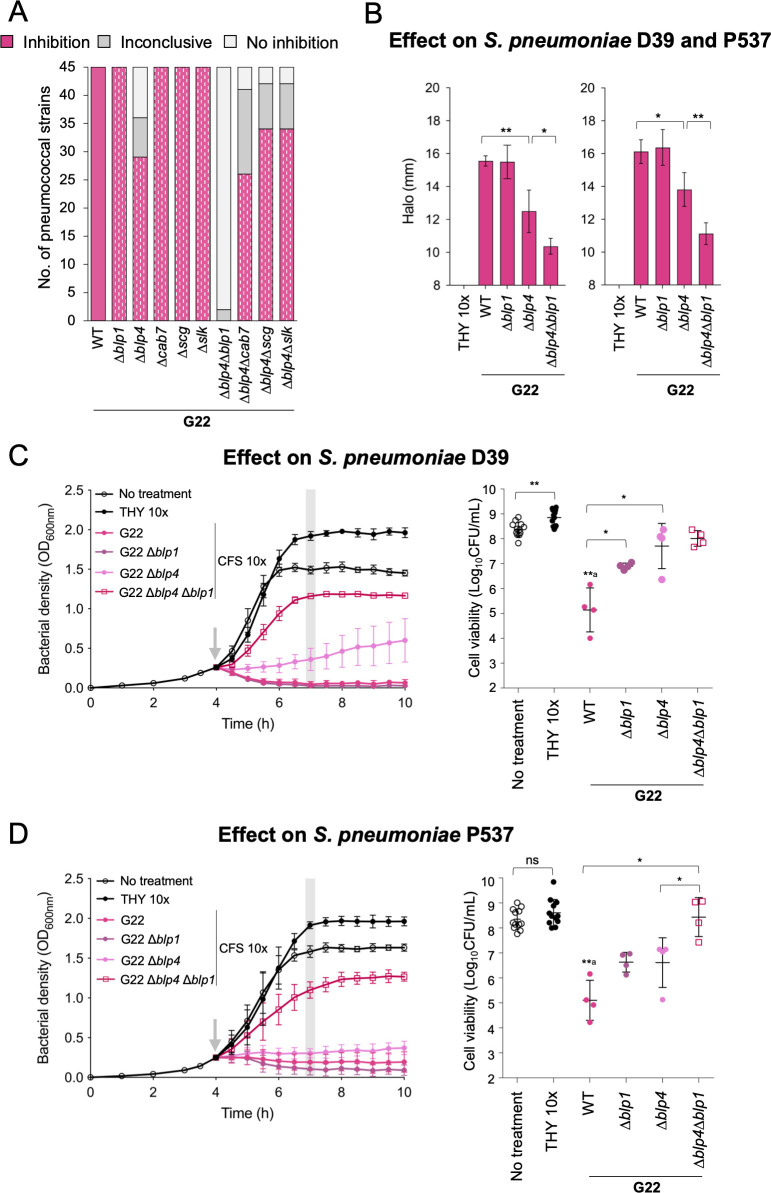
The anti-pneumococcal inhibitory activity of *S. mitis* strain G22 is driven by an additive activity of bacteriocin loci. (**A**) Strains G22 and its deletion mutants (marked with dashes within the colored bars) were tested for inhibitory activity against 45 *S*. *pneumoniae* strains (representatives of 23 serotypes and 42 multilocus sequence types) in agar overlay assays. Plates were inspected for growth inhibition halos around the stabbed strains (G22 and its mutants). Results were inspected independently by two researchers, in three independent experiments. (**B**) Well-diffusion assay using CFS 10× or THY 10× (control) against *S. pneumoniae* D39 (left) and P537 (right). After overnight incubation with CFS, plates were inspected for inhibition halos. The diameter of the inhibition halos was measured independently by two researchers in four independent experiments. Mean and standard deviation are represented for each condition. The inhibition halo diameter includes the well width of 8 mm. *P-value < 0.05 and **P-value < 0.01, Student’s *t*-test with Benjamini and Hochberg correction for FDR. (**C and D**) The effect of 10× CFS on planktonic growth of *S. pneumoniae* D39 (**C**) and P537 (**D**) was assessed. Early exponential phase cultures (OD_600nm_ of 0.3) of D39 and P537 were treated with 10% (vol/vol) of CFS 10×, THY 10×, or remained untreated (as indicated by the gray arrow) and OD_600nm_ was monitored every 30 min (left and middle panels). Gray bar indicates the time point in which an aliquot was taken for cell viability counts. Mean and standard deviation of at least four biological replicates are represented for each condition. Cell viability (CFU/mL) of D39 and P537 was assessed 3 h post-treatment (right panel). *P-value < 0.05 and **P-value < 0.01, Mann-Whitney *U* test with Benjamini and Hochberg correction for FDR; ^a^statistical comparison with treatment with THY 10×; ns, not significant.

Since deletion of a single bacteriocin-related locus from strains A22 to D22, F22, and G22 was not sufficient to abolish their anti-pneumococcal activity, we hypothesized that the inhibitory activity of bacteriocins from different loci could have an additive effect. To test this possibility, we focused on strain G22 since it showed a high transformation efficiency and, thus, was easier to manipulate. We used G22Δ*blp4*, a mutant that showed some (but not complete) loss of inhibitory activity, to construct double deletion mutants (G22Δ*blp4*Δ*blp1*, G22Δ*blp4*Δ*cab7*, G22Δ*blp4*Δ*scg*, and G22Δ*blp4*Δ*slk*). Deletion of both *blp1* and *blp4* completely abolished G22 inhibitory activity in the overlay assays, a result not observed with the corresponding single deletion mutants (Fig. 7A). This was also observed when the CFS of G22Δ*blp4*Δ*blp1* was tested against *S. pneumoniae* D39 and P537 growing in solid and liquid media (Fig. 7B through D). For other double mutants of G22, no significant additive effects were noted, albeit a small effect was noticed for the G22Δ*blp4*Δ*cab7* mutant (Fig. S8).

Overall, these results indicate that multiple loci may be necessary for complete inhibition of pneumococcal growth in a subset of the *S. mitis* strains.

### Strains A22 to G22 inhibit *S. pneumoniae* in multi-species biofilms

**Fig 8:**
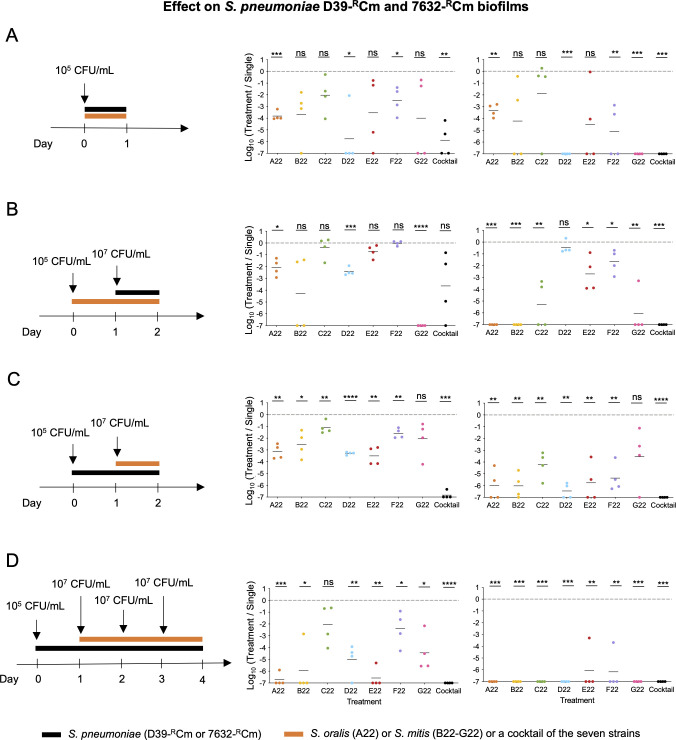
*S. oralis* strains A22 and *S. mitis* strains B22 to G22 inhibit *S. pneumoniae* in multi-species biofilms. The ability of strains A22 to G22, individually or in a seven-strain cocktail, to prevent or disrupt biofilms of *S. pneumoniae* strains D39-^R^Cm and 7632-^R^Cm was evaluated with both species inoculated in a 1:1 ratio. (A) Strains A22 to G22 were inoculated simultaneously with *S. pneumoniae* strains. Growth was evaluated after 24 h. (B) Strains A22 to G22 were grown for 24 h in biofilm prior to inoculation of *S. pneumoniae*. *S. pneumoniae* growth was evaluated after 24 h. (C and D) Strains A22 to G22 were added to pre-grown (24 h) *S. pneumoniae* biofilms as a single treatment or as a 3 day-treatment. *S. pneumoniae* growth was evaluated 24 h after treatment. In all experiments, single-strain biofilms of the corresponding strains were performed in parallel as control. *Y* axis represents the ratio between the bacterial density (CFU/mL) of *S. pneumoniae* biofilm after treatment with strains A22 to G22 and the bacterial density (CFU/mL) of *S. pneumoniae* biofilm without treatment. Four independent experiments were performed. *P < 0.05; **P < 0.01; ***P < 0.001; and ****P < 0.0001, ratio paired *t*-test; ns, not significant.

Having established that strains A22 to G22 inhibited the planktonic growth of *S. pneumoniae,* we next wanted to determine whether they could also prevent or otherwise interfere with the biofilm formation or stability. The biofilm mode of growth plays a critical role in the colonization of the URT. A key issue, then, was whether strains A22 to G22 were themselves able to form biofilms. We started by assessing this using an *in vitro* model previously validated (15, 16, 23). After 24 h, we observed a 2–3-log increase in the colony-forming unit (CFU) counts in the biofilm of all strains in comparison to the inoculum, confirming the capacity of these commensals to form biofilms (Fig. S9). We then set up dual-species biofilms to evaluate whether strains A22 to G22 were able to interfere with *S. pneumoniae* biofilm formation or stability at various stages. Two *S*. *pneumoniae* strains, D39-^R^Cm (serotype 2) and 7632-^R^Cm (serotype 15A), which are robust biofilm formers, were used (47). All the A22 to G22 strains interfered with *S. pneumoniae* biofilms, in several of the conditions tested (Fig. 8). For example, when inoculated at the same time with *S. pneumoniae*, strains A22, D22, and F22 individually inhibited *S. pneumoniae* biofilm formation (Fig. 8A). If added 24 h in advance, six of the strains (exception being D22) were able to severely affect the capacity of at least one *S*. *pneumoniae* to form biofilm (Fig. 8B). Finally, if added to a 24 h pre-existing biofilm of *S. pneumoniae*, all strains A22 to G22 had the capacity to significantly disrupt the biofilm of at least one *S*. *pneumoniae* strain (with A22 to C22 and E22 being able to disrupt both) (Fig. 8C). Notably, *S. oralis* A22 negatively affected *S. pneumoniae* biofilm in all conditions tested.

We next simulated a 3-day treatment where we added the strain of interest (A22 to G22) every 24 h to a pre-grown *S. pneumoniae* biofilm. Six of the seven strains (all but C22) were able to significantly inhibit *S. pneumoniae* D39-^R^Cm, and all were able to disrupt and, in most cases, even eliminate the biofilm of *S. pneumoniae* strain 7632-^R^Cm (Fig. 8D).

Finally, we tested the effect of adding a cocktail of the seven strains (A22 to G22). This was very effective in preventing *S. pneumoniae* biofilm formation: a 6-log decrease, compared to the no addition control, was observed in the case of D39-^R^Cm, and complete inhibition was observed in the case of 7632-^R^Cm (Fig. 8C).

Together, these results show the potential of strains A22 to G22 as agents to interfere with pneumococcal biofilm formation, a fundamental step of *in vivo* colonization.

## DISCUSSION

The potential of live bacterial strains with probiotic traits to be used as biotherapeutics for infectious diseases is being actively explored, focusing either on their action as modulators of the immune system or on their role as competitors of pathogenic microbes (15, 16, 23). Similar strategies have been investigated before, for example, to promote oral health (15, 16, 23). One strain of *S. oralis* is currently being used in a commercially available mouthwash to promote gingival recovery (15, 16, 23).

Here, we explored the potential of commensal streptococci to be used as probiotics or biotherapeutics to control *S. pneumoniae*, a major cause of respiratory infections worldwide.

We identified seven commensal streptococci (one *S*. *oralis* and six *S*. *mitis* strains among over 300 screened strains, named A22 to G22), with the potential to be used as anti-pneumococcal biotherapeutics or as sources of molecules, such as bacteriocins, with anti-pneumococcal activity. Notably, strains A22 to G22 can inhibit a wide variety of *S. pneumoniae* strains of multiple serotypes and genotypes. We found that they are equipped with a large and diverse repertoire of bacteriocin-encoding loci, in line with previous analyses of *Streptococcus* spp. (37, 38, 48, 49). Importantly, several of the bacteriocins and immunity proteins found in strains A22 to G22 are absent or are extremely rare in *S. pneumoniae*, potentially explaining their broad anti-pneumococcal activity. This was further supported for the *S. mitis* strains by the assays using cell-free supernatants, the strains themselves, or their corresponding bacteriocin deletion mutants. Indeed, over half of the bacteriocin loci identified in *S. mitis* strains contributed to the inhibitory activity in at least one of the conditions tested. The most striking example was the deletion of locus *blp1* in strains B22 and F22, which abolished their anti-pneumococcal activity. In general, however, the results pointed to the contribution of more than one locus to achieve broad inhibition, as observed for the cumulative effect of *blp1* and *blp4* in strain G22.

Interestingly, we were unable to demonstrate the role of bacteriocin loci of *S. oralis* strain A22 in the inhibition of pneumococci. No loss of inhibition was observed in the bacteriocin-deletion mutants, in line with the observation that protease treatment of cell-free supernatants of this strain did not destroy the still retained inhibitory effect. Together, these findings suggest that the inhibitory phenotype of *S. oralis* A22 is most probably due to secreted thermostable non-protein molecules or protease-resistant bacteriocins that may not have been identified in the genetic analysis, a subject that will require further investigation.

Of particular interest, strains A22 to G22 were able to prevent the formation of and disrupt pneumococcal biofilms, a proxy for nasopharyngeal colonization. The addition of commensal strains to pneumococcal biofilms for three consecutive days, simulating a 3-day repeated treatment, resulted in the highest degree of inhibition. Additionally, when the seven strains were combined in a single cocktail, pneumococcal biofilms were severely affected, indicating an added value of using more than one strain.

In conclusion, this study identified one *S*. *oralis* and six *S*. *mitis* strains with the potential to be used as live biotherapeutics, alone or in combination, to control *S. pneumoniae* colonization, a crucial step that precedes disease and transmission (12). Additionally, strains A22 to G22 could be used as platforms for the design of novel engineered strains with optimal characteristics for the same purpose. Finally, as the anti-pneumococcal activity was mostly associated with the production of bacteriocins, their direct use is a topic that deserves further attention (27–29) and that we are currently exploring. In a world grappling with the unprecedented threat of antibiotic-resistant bacteria, targeted approaches to control colonization and prevent disease caused by common pathobionts offer a promising solution and merit further exploration.

## MATERIALS AND METHODS

### Bacterial strains

Two bacterial collections were used: a collection of commensal non-pneumococcal streptococcal isolates used to screen for strains with anti-pneumococcal activity, and an *S. pneumoniae* collection against which the inhibitory activity of the commensal streptococci was evaluated.

The non-pneumococcal streptococcal strains (*n* = 313) were isolated from the upper respiratory tract of humans (children and adults) between 1999 and 2016 during carriage studies conducted by our laboratory (7, 31–33). Isolates were obtained from nasopharyngeal samples of healthy individuals not colonized with *S. pneumoniae* at the time of sampling and with no antimicrobial use in the month preceding sampling. Isolates were initially characterized based on the observation of hemolysis, colony morphology, optochin susceptibility, bile solubility, and/or the presence of a copy of a typical *S. pneumoniae lytA* (50).

The *S. pneumoniae* strains (*n* = 230) were recovered from the nasopharyngeal swabs of children up to 6 years old between 1999 and 2016 during carriage studies conducted by our laboratory: 153 strains were selected to represent epidemiologically relevant serotypes (7, 31), and 77 were representative of diverse BIRs (35). Strains were previously characterized based on serotyping and MLST (Table S1) (7, 31, 35). In addition, *S. pneumoniae* strains P537, P133, and D39 were used as controls in overlay and/or supernatant assays. Strain P537 is a serotype 6A strain from which the entire bacteriocin-like peptide (*blp*) locus was deleted, being susceptible to bacteriocins of *blp* type (34). It was used as a negative control for inhibitory activity in overlay assays and as a susceptible indicator of inhibition in both overlay and supernatant assays. Strain P133 is a serotype 6A strain with an active *blp* locus (34). It was used as a positive control for inhibitory activity in the overlay assays. Strain D39 is a laboratory strain of serotype 2 easily manipulated in lab conditions (51). D39 was used as an indicator strain in the supernatant assays. Finally, *S. pneumoniae* strains D39-^R^Cm and 7632-^R^Cm (serotype 15A) were used in biofilm and overlay assays. These strains are resistant to chloramphenicol (due to the insertion of P*_hlpA_-hlpA-gfp*_Camr construct) (52), enabling their easy selection following co-culture experiments (47).

### Inhibition overlay assays

Inhibition overlay assays were done as described before (34, 53). Controls and test strains were grown in Todd-Hewitt broth (Bacto, BD) supplemented with 0.5% yeast extract (Fisher Scientific) (THY) at 37°C until an OD_600nm_ of 0.5 was reached. Glycerol was added to the cultures at a final concentration of 15%, and the mixtures were transferred to a 96-well plate. Then, strains were plated using a replica plater on 20 mL of Tryptic Soy agar supplemented with 5% sheep blood (BBL Stacker Plates, BD) (BA) and incubated overnight at 37°C in 5% CO_2_. On the following day, the growth in the BA plates was collected with a replica plater and stabbed onto Tryptic Soy broth (Bacto, BD) with 1.5% agar (Fisher Scientific) (TSAgar) plates supplemented with 4,750 U of catalase (Sigma-Aldrich). The stabbed plates were incubated for 6 h at 37°C in 5% CO_2_.

Overlay strains were grown in THY at 37°C until an OD_600nm_ of 0.5 was reached. A mixture containing 200 µL of the overlay strain, 4,750 U of catalase, 5 mL of warm THY, and 3 mL of molten TSAgar was gently dispensed on top of the stabbed TSAgar plates. After solidification of the overlay layer, the plates were incubated overnight at 37°C in 5% CO_2_. On the following day, plates were inspected for growth inhibition halos around the stabbed strain—indicating inhibitory activity of the test strain toward the overlay strain. At least three independent experiments were done for each overlay strain. Test strains were considered inhibitory toward the overlay strain if inhibition halos were observed in at least two of three independent experiments.

### Whole-genome sequencing

Genomes of the seven strains of interest (A22 to G22) were sequenced by Illumina Miseq platform and Oxford Nanopore MinION technology with a minimum coverage of 100× for each method. Library preparation and sequencing were done at the Genomics Unit of Instituto Gulbenkian de Ciência (Oeiras, Portugal). Illumina paired-end reads were checked for quality and trimmed with default parameters using the Qiagen CLC Genomics Workbench software version 9.5.1 (Qiagen). MinION reads were checked for quality and filtered using the Filtlong tool version 0.2.0. Hybrid assemblies using both Illumina and MinION reads were done using the Unicycler pipeline version 0.4.8-beta (54), and the final assemblies were visualized using the Bandage tool version 0.8.1 (55). The consensus sequences were extracted and annotated with the Prokka pipeline version 1.13.3 (last accessed on 18 September 2020) (56).

### Multilocus sequence analysis for viridans group streptococci

MLSA for species identification of strains A22 to G22 was done as described elsewhere (36). DNA sequences were extracted from genome sequencing data and concatenated in-frame into a 3,063 bp fasta file for each strain. Phylogenetic analysis of the concatenated sequences in comparison with the eMLSA database (consisting of 413 strains of 25 viridans streptococcal species plus 14 strains of unknown/uncertain species) (55) was performed using CLC Genomics Workbench (Qiagen). A neighbor-joining phylogenetic tree was generated using the Jukes-Cantor model for nucleotide distance measure, and bootstrap analysis was performed based on 500 replicates.

### Preparation of cell-free supernatants

Strains A22 to G22 were grown in THY at 37°C until the early stationary phase. Cultures were centrifuged at 5,000 × *g* for 10 min at 4°C, and the CFSs were collected and filtered with a 0.2 µm filter. CFSs were concentrated 10-fold (10× CFS). Concentration was achieved by water evaporation under vacuum using the Acid-Resistant CentriVap Vacuum Concentrator (Labconco) to allow concentration of all components by the same factor. When indicated, 10× CFSs from wild-type strains were heat- and protease-treated. For heat treatment, samples were incubated at 37°C, 45°C, 60°C, and 75°C for 1.5 h, at 95°C for 1 h, and at 121°C for 20 min (the latter mimicking sterilizing conditions). For protease treatment, samples were incubated with 1 mg/mL proteinase K (Roche Diagnostics) at 37°C for 3 h. Untreated and treated supernatants were tested for inhibitory activity. As a control, THY was incubated, treated, and 10× concentrated in parallel with all samples.

### Well-diffusion assays

*S. pneumoniae* D39 and P537 were grown in THY at 37°C until an OD_600nm_ of 0.5 was reached. A mixture containing 750 µL of D39 or P537 culture, 7,125 U of catalase, 8 mL of warm THY, and 21 mL of molten TSAgar was dispensed in a Petri dish. After solidification, wells were done in the agar using the top of 1 mL-pipette tips, and 200 µL of 10× CFS to be tested (of strains A22 to G22 or its derivatives) was poured inside the well (one supernatant per well). As a negative control, 10× THY was used. The plates were incubated overnight at 37°C in 5% CO_2_. On the following day, the plates were inspected for growth inhibition halos around the well—indicating inhibitory activity of the supernatant towards the bacterial strain. At least three independent experiments were done for each supernatant.

### Growth curves and cell viability

*S. pneumoniae* D39 and P537 were grown in THY at 37°C until an OD_600nm_ of 0.3 was reached. Cultures were split into 3 mL aliquots and treated with 300 µL of 10× CFS of strains A22 to G22 or 10× THY. The OD_600nm_ was monitored every 30 min.

In addition, the cell viability of pneumococcal cultures treated with CFS of strains A22 to G22 was determined. One hundred microliter aliquots were taken 3 h after CFS treatment and serially diluted in PBS. For each dilution, three 20 µL drops were plated in BA and incubated overnight at 37°C in a 5% CO_2_ atmosphere. On the following day, colony-forming units were counted.

### *In silico* search for bacteriocin-related loci

The annotated assembled genomes were submitted to the tools BAGEL4 (57) and antiSMASH version 5.0 (58). The identified putative bacteriocin and immunity protein sequences were used as input motifs in MAST-MEME Suite version 5.4.1 to search for additional bacteriocin loci (59). The outputs from all tools were manually examined, cross-examined, and combined, allowing us to establish more confident gene clusters. Small ORFs not automatically annotated with the Prokka pipeline were searched using the “find open reading frame” (any start codon) tool from CLC Genomics Workbench (Qiagen), and their putative function was examined by BLASTp search against the NCBI database and/or the UniProt database. Transmembrane helical domains were predicted for putative immunity proteins using the tool TMHMM version 2.0 (60). Streptococcin loci were defined as those containing lactococcin 972-like bacteriocins, as previously described (38). Lantibiotic loci were defined as those containing modification enzymes within the locus (39).

To determine the similarity among bacteriocins and immunity proteins, respectively, alignments were made using all putative bacteriocins or immunity proteins found among strains A22 to G22 and known ones from *S. pneumoniae*. Alignments were done using CLC Genomics Workbench (Qiagen) with default parameters. Proteins sharing 100% length and 100% sequence identity were considered the same. Proteins sharing ≥90% length and ≥80% sequence identity were considered variants of the same protein. The above criteria were applied to the full-length protein sequences except for bacteriocins from bacteriocin-like peptide (*blp*), competence-associated bacteriocin (*cab*), and mitilancidin (*mld*) loci, for which mature sequences were considered. When exceptions to the above criteria were observed (such as truncated versions of bacteriocins previously described or identification of proteins for which the percentage of shared length and sequence identity was just below the thresholds defined for variants), the protein sequences were manually inspected to assess similarity and determine which regions were contributing to the lower coverage and/or identity.

To determine the prevalence of bacteriocins and immunity proteins found in strains A22 to G22 among *S. pneumoniae*, *S. mitis*, and *S. oralis*, a search was performed using a collection of 7,548 pneumococcal, 322 *S*. *mitis,* and 108 *S*. *oralis* genomes deposited at the PubMLST Oral *Streptococcus* spp. Database. The pneumococcal collection was previously selected for quality by Melissa Jansen van Rensburg and Angela Brueggemann (41). Briefly, a tBLASTn of putative bacteriocin and immunity protein sequences from strains A22 to G22 was done against all genomes. The results were manually examined and cutoff values for prevalence were postulated at 100% query length and 100% sequence identity (strict criteria) or ≥90% query length and ≥80% sequence identity (relaxed criteria).

### Characterization of bacteriocin loci regulatory regions

*In silico* characterization of putative promoters and terminators of the bacteriocin loci genes was done. For promoter analysis, the upstream regions (250 bp) of the bacteriocin genes were pulled, and shared DNA motifs for putative binding sequences of transcriptional regulators were searched with MEME software (61). Additionally, binding sites for BlpR (NYAATTCAAGANGTTTYRATG-ACAATTCAAG(NN)ATTTGRANN) (43), ComE (TNYWVTTBRGR-[N_11_]-ACADTTGAGR) (43), SigX (TTT-[N]-HNCGAATA) (42, 43), and RpoD (TTGACA-[N_15-19_]-TATAAT) (62) were searched. Inferred binding sites for catabolite-responsive element (NNDNNWNNVNDNNHNN) and *boxABC* (BNVBVYY-[N_4_]-TACTHYBHNRRAMTHNNWVTDYMMDYNDBDKHVNHNYHNNHYYVNHNYVNNN NNNNNNW; BNDDHNNBDHHRVYYHYVHYHNVNHVNNYHVARNHNVTD-[N_2_]-NBTYDVN; NHNNHHDNDNHHVNYYDBGGHTWRBTYNNN-[N_8_]-NWWBNNTYYYYRYHBWNNDNNN) from *S. pneumoniae* D39 strain were also searched (62). We used Find Individual Motifs Occurrences (FIMO) to scan for the specific binding site of each transcriptional factor at the bacteriocin loci of interest (63). A cutoff false-discovery rate (*q*) value of 0.05 was used. All binding sites detected were scrutinized with DNA alignment tools available at CLC Genomics Workbench software using default parameters (Qiagen) (64), with the consensus binding logos created via WebLogo (65). The identification of Rho-independent terminators was done using ARNold, FindTerm, and mFold (66, 67).

### qRT-PCR

Strains A22 to G22 were grown in C + Y_YB_ (68) at 37°C with 5% CO_2_ until an OD_600nm_ of 0.04 was reached. Cultures were divided into two, with one being treated with 300 nM of synthetic cognate BlpC (Mimotopes, Australia), and the other half kept untreated (Table S6). Cultures were incubated for 5 min and immediately put on ice. Total RNA was extracted with the RNeasy kit (Qiagen) and treated with DNase I (Qiagen) to remove genomic DNA, following the manufacturer’s recommendations. For qRT-PCR, cDNAs were obtained with the cDNA Synthesis Kit (Bioline), and the resulting product was used for qRT-PCR with SensiFAST SYBR Kit (Bioline). Real-time quantitative PCR was carried out on three independent biological replicates in a CFX96 Touch real-time PCR detection system (Bio-Rad). Primers’ sequences are described in Table S6. Data were analyzed with the software Bio-Rad CFX (Bio-Rad). Housekeeping *gyrA* gene was used to normalize the results. The fold change in expression compared to untreated condition was determined by the 2^−ΔΔCT^ method (69).

### Construction of plasmids pKan, pChl, and pJB01

Plasmid pKan containing a *lox66-P3-KanR-lox71* cassette was synthesized (TwistBioscience). For the construction of pChl, the backbone of pKan and the ChlR marker were amplified (Table S6). Fragments were ligated with T4 DNA Ligase (ThermoFisher) and transformed into *E. coli* DH5α, generating plasmid pChl containing *lox66-P3-ChlR-lox71*. These two plasmids were used for the construction of bacteriocin-deficient mutants (described below) expressing resistance to either Kan or Chl.

To construct pJB01, the *cas9* of plasmid pDS05 (70) was removed by digestion with HindIII (NEB) and re-ligation. The *cre* and *spec* genes were PCR amplified from plasmid pDR244 (71) (Table S6), and the fragments were digested with BsaI (NEB). The fragment was ligated with BsaI-cut pDS05 Δ*cas9* generating plasmid pJB01 (Fig. S10). pJB01 was used to remove the antimicrobial marker, when possible, from the bacteriocin-deficient mutants.

### Generation of bacteriocin-deficient mutants

Deletion mutants of bacteriocin-immunity regions (containing all predicted bacteriocins and immunity proteins for that locus) were constructed for *blp*, *cab*, and streptococcin (lactococcin 972-like) loci. In the case of lantibiotic loci, only the bacteriocin genes were deleted. F22 Δ*blp1* was generated as previously described (72). The remaining mutants were generated by allelic replacement with an antibiotic resistance gene flanked by *lox66* and *lox71* sites. Two fragments flanking the genes to be deleted were amplified from genomic DNA, and the *lox66-P3-KanR-lox71* or *lox66-P3-ChlR-lox71* cassette was amplified from pKan or pChl, respectively. Fragments were purified using the Zymoclean Gel DNA Recovery Kit (Zymo Research) and ligated by Gibson Assembly (NEB) or SOE-PCR. Nested primers were used to amplify the desired product. Primers used to construct the fragments for transformation are listed in Table S6. For transformation, strains were grown in C + Y_YB_ medium without shaking at 37°C until an OD_600nm_ of 0.5. Cultures were diluted 1:100 in fresh C + Y_YB_ and grown until an OD_600nm_ of 0.04–0.1. At that point, cultures were supplemented with 150 ng/mL of DNA and 300 nM of cognate CSP (NZYTech, >85% purity) and further incubated for 4 h (S. *mitis*) or 2 h (*S. oralis*). Transformants were selected in BA with 300 µg/mL of kanamycin (Kan) or 4 µg/mL of chloramphenicol (Chl). Transformants were confirmed by PCR.

Bacteriocin-deficient markerless mutants were subsequently obtained by removing antibiotic resistance genes upon transformation with pJB01 (temperature sensitive) (Fig. S10). The Cre encoded in pJB01 recognizes the flanking *lox66* and *lox71* sites and excises the resistance marker, generating a single *lox72* site, which is not efficiently recognized by Cre in further manipulations (73). Strains were transformed with pJB01 as described above, with minor changes. After a 10 min incubation with CSP at 37°C, 1 µg of plasmid was added, and cultures were shifted to 30°C for 4 h. Cultures were plated in BA with 200 µg/mL of spectinomycin and incubated at 30°C. Single colonies were plated in BA with no antibiotics, BA with 300 µg/mL of Kan or 4 µg/mL of Chl, and BA with 200 µg/mL of Spec. Colonies that only grew in BA with no antibiotics (putative transformants of interest) were confirmed by PCR.

### Biofilms

Single and dual-species biofilms were performed in an abiotic surface with some changes regarding what was previously described (47). Strains were grown in THY until the mid-exponential phase (OD_600nm_ ~ 0.5). Each strain was diluted to 10^5^ CFU/mL in 2.5 mL of THY supplemented with catalase (1,600 U/mL), inoculated on 24-well plates, and incubated at 34°C in 5% CO_2_. Dual-species biofilms were grown in a 1:1 ratio and included an *S. pneumoniae* strain (D39-^R^Cm and 7632-^R^Cm) and one commensal strain (A22, B22, C22, D22, E22, F22, or G22) or a cocktail of the seven strains. Four experimental designs were tested: (i) *S. pneumoniae* and commensal strain inoculated at the same time in a mixture; (ii) commensal strain inoculated 24 h before *S. pneumoniae* strain; (iii) *S. pneumoniae* inoculated 24 h before the commensal strain; and (iv) a 24 h *S*. *pneumoniae* biofilm inoculated three times (1/day) with a commensal strain. In experiments (ii), (iii), and (iv), at 24 h, 1.25 mL of spent medium was carefully removed and a fresh culture of *S. pneumoniae* or commensal strain (at 10^7^ CFU/mL) or the cocktail in 1.25 mL of fresh medium was added avoiding disturbance of the pre-formed biofilm. In experiment (iv), the procedure was repeated at 48 and 72 h. As controls, single biofilms of the strains being tested were done in parallel using the same starter inoculum and replacing the spent medium with fresh medium at the pre-determined times. Biofilms were incubated and, after 24 h of the last inoculation, supernatants were carefully removed, and biofilms were resuspended in 500 µL of PBS. Serial dilutions for viability assessment were plated in parallel in BA and BA supplemented with 8 µg/mL of chloramphenicol for discrimination between strains. CFUs were counted after overnight incubation at 37°C in a 5% CO_2_-enriched atmosphere.

### Statistical analyses

The two-tailed unpaired Mann-Whitney *U* test with Benjamini and Hochberg correction for FDR was used for comparisons of *S. pneumoniae* viability treated with CFS of strains A22 to G22. Student’s *t*-test with Benjamini and Hochberg correction for FDR was used to compare the effect of CFS on well-diffusion assays. The ratio paired *t*-test was used for the statistical analyses of multi-species biofilms. A *P*-value ≤ 0.05 was considered significant. All statistical calculations were done using GraphPad Prism 9.0 (GraphPad Software Inc., La Jolla, CA, USA).

## Data Availability

The raw reads and completed genomes of the seven strains were deposited in the ENA database under the study accession number PRJEB75690: *Streptococcus oralis* A22 (ERS19845126), *Streptococcus mitis* B22 (ERS19845127), *Streptococcus mitis* C22 (ERS19845128), *Streptococcus mitis* D22 (ERS19845129), *Streptococcus mitis* E22 (ERS19845130), *Streptococcus mitis* F22 (ERS19845131), and *Streptococcus mitis* G22 (ERS19845132).

## SUPPLEMENTAL MATERIAL

Supplemental figures aem.01336-24-s0001.pdf Figures S1 to S10.

Supplemental tables aem.01336-24-s0002.xlsx Tables S1 to S7.

ASM does not own the copyrights to Supplemental Material that may be linked to, or accessed through, an article. The authors have granted ASM a non-exclusive, world-wide license to publish the Supplemental Material files. Please contact the corresponding author directly for reuse.

## References

[1] Wahl B, O’Brien KL, Greenbaum A, Majumder A, Liu L, Chu Y, Lukšić I, Nair H, McAllister DA, Campbell H, Rudan I, Black R, Knoll MD. 2018. Burden of Streptococcus pneumoniae and Haemophilus influenzae type b disease in children in the era of conjugate vaccines: global, regional, and national estimates for 2000-15. Lancet Glob Health 6:e744–e757. doi:10.1016/S2214-109X(18)30247-X 29903376 PMC6005122

[2] Drijkoningen JJC, Rohde GGU. 2014. Pneumococcal infection in adults: burden of disease. Clin Microbiol Infect 20 Suppl 5:45–51. doi:10.1111/1469-0691.12461 24313448

[3] Ganaie F, Saad JS, McGee L, van Tonder AJ, Bentley SD, Lo SW, Gladstone RA, Turner P, Keenan JD, Breiman RF, Nahm MH. 2020. A new pneumococcal capsule type, 10D, is the 100th Serotype and has a large cps fragment from an oral streptococcus. MBio 11:e00937-20. doi:10.1128/mBio.00937-20 32430472 PMC7240158

[4] Ganaie F, Maruhn K, Li C, Porambo RJ, Elverdal PL, Abeygunwardana C, van der Linden M, Duus JØ, Sheppard CL, Nahm MH. 2021. Structural, genetic, and serological elucidation of Streptococcus pneumoniae serogroup 24 serotypes: discovery of a new serotype, 24C, with a variable capsule structure. J Clin Microbiol 59:e0054021. doi:10.1128/JCM.00540-21 33883183 PMC8218768

[5] Essink B, Sabharwal C, Cannon K, Frenck R, Lal H, Xu X, Sundaraiyer V, Peng Y, Moyer L, Pride MW, Scully IL, Jansen KU, Gruber WC, Scott DA, Watson W. 2022. Pivotal phase 3 randomized clinical trial of the safety, tolerability, and immunogenicity of 20-valent pneumococcal conjugate vaccine in adults aged ≥18 Years. Clin Infect Dis 75:390–398. doi:10.1093/cid/ciab990 34940806 PMC9427137

[6] Weinberger DM, Malley R, Lipsitch M. 2011. Serotype replacement in disease after pneumococcal vaccination. Lancet 378:1962–1973. doi:10.1016/S0140-6736(10)62225-8 21492929 PMC3256741

[7] Félix S, Handem S, Nunes S, Paulo AC, Candeias C, Valente C, Simões AS, Almeida ST, Tavares DA, Brito-Avô A, de Lencastre H, Sá-Leão R. 2021. Impact of private use of the 13-valent pneumococcal conjugate vaccine (PCV13) on pneumococcal carriage among Portuguese children living in urban and rural regions. Vaccine (Auckl) 39:4524–4533. doi:10.1016/j.vaccine.2021.06.035 34183206

[8] Cherazard R, Epstein M, Doan TL, Salim T, Bharti S, Smith MA. 2017. Antimicrobial resistant Streptococcus pneumoniae: prevalence, mechanisms, and clinical implications. Am J Ther 24:e361–e369. doi:10.1097/MJT.0000000000000551 28430673

[9] Langdon A, Crook N, Dantas G. 2016. The effects of antibiotics on the microbiome throughout development and alternative approaches for therapeutic modulation. Genome Med 8:39. doi:10.1186/s13073-016-0294-z 27074706 PMC4831151

[10] Mayor A, Chesnay A, Desoubeaux G, Ternant D, Heuzé-Vourc’h N, Sécher T. 2021. Therapeutic antibodies for the treatment of respiratory tract infections—current overview and perspectives. Vaccines (Basel) 9:151. doi:10.3390/vaccines9020151 33668613 PMC7917879

[11] Simell B, Auranen K, Käyhty H, Goldblatt D, Dagan R, O’Brien KL, Pneumococcal Carriage Group. 2012. The fundamental link between pneumococcal carriage and disease. Expert Rev Vaccines 11:841–855. doi:10.1586/erv.12.53 22913260

[12] Weiser JN, Ferreira DM, Paton JC. 2018. Streptococcus pneumoniae: transmission, colonization and invasion. Nat Rev Microbiol 16:355–367. doi:10.1038/s41579-018-0001-8 29599457 PMC5949087

[13] Vidal JE, Wier MN, A. Angulo-Zamudio U, McDevitt E, Jop Vidal AG, Alibayov B, Scasny A, Wong SM, Akerley BJ, McDaniel LS. 2021. Prophylactic inhibition of colonization by streptococcus pneumoniae with the secondary bile acid metabolite deoxycholic acid. Infect Immun 89:e0046321. doi:10.1128/IAI.00463-21 34543118 PMC8594607

[14] Alreja AB, Appel AE, Zhu JC, Riley SP, Gonzalez-Juarbe N, Nelson DC. 2024. SP-CHAP, an endolysin with enhanced activity against biofilm pneumococci and nasopharyngeal colonization. MBio 15:e0006924. doi:10.1128/mbio.00069-24 38470268 PMC11005408

[15] Cunningham M, Azcarate-Peril MA, Barnard A, Benoit V, Grimaldi R, Guyonnet D, Holscher HD, Hunter K, Manurung S, Obis D, Petrova MI, Steinert RE, Swanson KS, van Sinderen D, Vulevic J, Gibson GR. 2021. Shaping the future of probiotics and prebiotics. Trends Microbiol 29:667–685. doi:10.1016/j.tim.2021.01.003 33551269

[16] Yang B, Fang D, Lv Q, Wang Z, Liu Y. 2021. Targeted therapeutic strategies in the battle against pathogenic bacteria. Front Pharmacol 12:673239. doi:10.3389/fphar.2021.673239 34054548 PMC8149751

[17] Geldart KG, Kommineni S, Forbes M, Hayward M, Dunny GM, Salzman NH, Kaznessis YN. 2018. Engineered E. coli Nissle 1917 for the reduction of vancomycin-resistant Enterococcus in the intestinal tract. Bioeng Transl Med 3:197–208. doi:10.1002/btm2.10107 30377660 PMC6195901

[18] Hwang IY, Koh E, Wong A, March JC, Bentley WE, Lee YS, Chang MW. 2017. Engineered probiotic Escherichia coli can eliminate and prevent Pseudomonas aeruginosa gut infection in animal models. Nat Commun 8:15028. doi:10.1038/ncomms15028 28398304 PMC5394271

[19] Nakatsuji T, Hata TR, Tong Y, Cheng JY, Shafiq F, Butcher AM, Salem SS, Brinton SL, Rudman Spergel AK, Johnson K, Jepson B, Calatroni A, David G, Ramirez-Gama M, Taylor P, Leung DYM, Gallo RL. 2021. Development of a human skin commensal microbe for bacteriotherapy of atopic dermatitis and use in a phase 1 randomized clinical trial. Nat Med 27:700–709. doi:10.1038/s41591-021-01256-2 33619370 PMC8052297

[20] Andaloro C, Santagati M, Stefani S, La Mantia I. 2019. Bacteriotherapy with Streptococcus salivarius 24SMB and Streptococcus oralis 89a oral spray for children with recurrent Streptococcal pharyngotonsillitis: a randomized placebo-controlled clinical study. Eur Arch Otorhinolaryngol 276:879–887. doi:10.1007/s00405-019-05346-3 30767047

[21] Tagg JR. 2004. Prevention of streptococcal pharyngitis by anti-Streptococcus pyogenes bacteriocin-like inhibitory substances (BLIS) produced by Streptococcus salivarius. Indian J Med Res 119 Suppl:13–16. 15232154

[22] Ferrer MD, López-López A, Nicolescu T, Perez-Vilaplana S, Boix-Amorós A, Dzidic M, Garcia S, Artacho A, Llena C, Mira A. 2020. Topic application of the probiotic Streptococcus dentisani improves clinical and microbiological parameters associated with oral health. Front Cell Infect Microbiol 10:465. doi:10.3389/fcimb.2020.00465 32984080 PMC7488176

[23] Debnath N, Kumar A, Yadav AK. 2022. Probiotics as a biotherapeutics for the management and prevention of respiratory tract diseases. Microbiol Immunol 66:277–291. doi:10.1111/1348-0421.12980 35462444

[24] Panigrahi P, Parida S, Nanda NC, Satpathy R, Pradhan L, Chandel DS, Baccaglini L, Mohapatra A, Mohapatra SS, Misra PR, Chaudhry R, Chen HH, Johnson JA, Morris JG, Paneth N, Gewolb IH. 2017. A randomized synbiotic trial to prevent sepsis among infants in rural India. Nature New Biol 548:407–412. doi:10.1038/nature23480 28813414

[25] Kilian M, Riley DR, Jensen A, Brüggemann H, Tettelin H. 2014. Parallel evolution of Streptococcus pneumoniae and Streptococcus mitis to pathogenic and mutualistic lifestyles. MBio 5:e01490-14. doi:10.1128/mBio.01490-14 25053789 PMC4120201

[26] Kilian M, Poulsen K, Blomqvist T, Håvarstein LS, Bek-Thomsen M, Tettelin H, Sørensen UBS. 2008. Evolution of Streptococcus pneumoniae and its close commensal relatives. PLoS One 3:e2683. doi:10.1371/journal.pone.0002683 18628950 PMC2444020

[27] Heilbronner S, Krismer B, Brötz-Oesterhelt H, Peschel A. 2021. The microbiome-shaping roles of bacteriocins. Nat Rev Microbiol 19:726–739. doi:10.1038/s41579-021-00569-w 34075213

[28] Cotter PD, Ross RP, Hill C. 2013. Bacteriocins - a viable alternative to antibiotics? Nat Rev Microbiol 11:95–105. doi:10.1038/nrmicro2937 23268227

[29] Rea MC, Dobson A, O’Sullivan O, Crispie F, Fouhy F, Cotter PD, Shanahan F, Kiely B, Hill C, Ross RP. 2011. Effect of broad- and narrow-spectrum antimicrobials on Clostridium difficile and microbial diversity in a model of the distal colon. Proc Natl Acad Sci U S A 108 Suppl 1:4639–4644. doi:10.1073/pnas.1001224107 20616009 PMC3063588

[30] Zipperer A, Konnerth MC, Laux C, Berscheid A, Janek D, Weidenmaier C, Burian M, Schilling NA, Slavetinsky C, Marschal M, Willmann M, Kalbacher H, Schittek B, Brötz-Oesterhelt H, Grond S, Peschel A, Krismer B. 2016. Human commensals producing a novel antibiotic impair pathogen colonization. Nature New Biol 535:511–516. doi:10.1038/nature18634 27466123

[31] Nunes S, Félix S, Valente C, Simões AS, Tavares DA, Almeida ST, Paulo AC, Brito-Avô A, de Lencastre H, Sá-Leão R. 2016. The impact of private use of PCV7 in 2009 and 2010 on serotypes and antimicrobial resistance of Streptococcus pneumoniae carried by young children in portugal: comparison with data obtained since 1996 generating a 15-year study prior to PCV13 introduction. Vaccine (Auckl) 34:1648–1656. doi:10.1016/j.vaccine.2016.02.045 26920470

[32] Almeida ST, Paulo AC, Froes F, de Lencastre H, Sá-Leão R. 2021. Dynamics of pneumococcal Carriage in Adults: A New Look at an Old Paradigm. J Infect Dis 223:1590–1600. doi:10.1093/infdis/jiaa558 32877517

[33] Almeida ST, Nunes S, Santos Paulo AC, Valadares I, Martins S, Breia F, Brito-Avô A, Morais A, de Lencastre H, Sá-Leão R. 2014. Low prevalence of pneumococcal carriage and high serotype and genotype diversity among adults over 60 years of age living in Portugal. PLoS One 9:e90974. doi:10.1371/journal.pone.0090974 24604030 PMC3946249

[34] Son MR, Shchepetov M, Adrian PV, Madhi SA, de Gouveia L, von Gottberg A, Klugman KP, Weiser JN, Dawid S. 2011. Conserved mutations in the pneumococcal bacteriocin transporter gene, blpA, result in a complex population consisting of producers and cheaters. MBio 2:e00179-11. doi:10.1128/mBio.00179-11 21896678 PMC3171984

[35] Valente C, Dawid S, Pinto FR, Hinds J, Simões AS, Gould KA, Mendes LA, de Lencastre H, Sá-Leão R. 2016. The blp locus of Streptococcus pneumoniae plays a limited role in the selection of strains that can cocolonize the human nasopharynx. Appl Environ Microbiol 82:5206–5215. doi:10.1128/AEM.01048-16 27316956 PMC4988185

[36] Bishop CJ, Aanensen DM, Jordan GE, Kilian M, Hanage WP, Spratt BG. 2009. Assigning strains to bacterial species via the internet. BMC Biol 7:3. doi:10.1186/1741-7007-7-3 19171050 PMC2636762

[37] Bogaardt C, van Tonder AJ, Brueggemann AB. 2015. Genomic analyses of pneumococci reveal a wide diversity of bacteriocins - including pneumocyclicin, a novel circular bacteriocin. BMC Genomics 16:554. doi:10.1186/s12864-015-1729-4 26215050 PMC4517551

[38] Rezaei Javan R, van Tonder AJ, King JP, Harrold CL, Brueggemann AB. 2018. Genome sequencing reveals a large and diverse repertoire of antimicrobial peptides. Front Microbiol 9:2012. doi:10.3389/fmicb.2018.02012 30210481 PMC6120550

[39] McAuliffe O, Ross RP, Hill C. 2001. Lantibiotics: structure, biosynthesis and mode of action. FEMS Microbiol Rev 25:285–308. doi:10.1111/j.1574-6976.2001.tb00579.x 11348686

[40] Bushin LB, Clark KA, Pelczer I, Seyedsayamdost MR. 2018. Charting an unexplored streptococcal biosynthetic landscape reveals a unique peptide cyclization motif. J Am Chem Soc 140:17674–17684. doi:10.1021/jacs.8b10266 30398325

[41] Abdullah IT, Ulijasz AT, Girija UV, Tam S, Andrew P, Hiller NL, Wallis R, Yesilkaya H. 2022. Structure-function analysis for the development of peptide inhibitors for a Gram-positive quorum sensing system. Mol Microbiol 117:1464–1478. doi:10.1111/mmi.14921 35575437 PMC9233744

[42] Salvadori G, Junges R, Åmdal HA, Chen T, Morrison DA, Petersen FC. 2018. High-resolution profiles of the Streptococcus mitis CSP signaling pathway reveal core and strain-specific regulated genes. BMC Genomics 19:453. doi:10.1186/s12864-018-4802-y 29898666 PMC6001120

[43] Slager J, Aprianto R, Veening JW. 2019. Refining the pneumococcal Competence regulon by RNA sequencing. J Bacteriol 201:e00780-18. doi:10.1128/JB.00780-18 30885934 PMC6560143

[44] Kjos M, Miller E, Slager J, Lake FB, Gericke O, Roberts IS, Rozen DE, Veening JW. 2016. Expression of Streptococcus pneumoniae bacteriocins is induced by antibiotics via regulatory interplay with the competence system. PLoS Pathog 12:e1005422. doi:10.1371/journal.ppat.1005422 26840404 PMC4739728

[45] Knutsen E, Johnsborg O, Quentin Y, Claverys J-P, Håvarstein LS. 2006. BOX elements modulate gene expression in Streptococcus pneumoniae: impact on the fine-tuning of competence development. J Bacteriol 188:8307–8312. doi:10.1128/JB.00850-06 16997972 PMC1698192

[46] Dawid S, Roche AM, Weiser JN. 2007. The blp bacteriocins of Streptococcus pneumoniae mediate intraspecies competition both in vitro and in vivo. Infect Immun 75:443–451. doi:10.1128/IAI.01775-05 17074857 PMC1828380

[47] Valente C, Cruz AR, Henriques AO, Sá-Leão R. 2021. Intra-Species Interactions in Streptococcus pneumoniae Biofilms. Front Cell Infect Microbiol 11:803286. doi:10.3389/fcimb.2021.803286 35071049 PMC8767070

[48] Vertillo Aluisio G, Spitale A, Bonifacio L, Privitera GF, Stivala A, Stefani S, Santagati M. 2022. Streptococcus salivarius 24SMBc genome analysis reveals new biosynthetic gene clusters involved in antimicrobial effects on Streptococcus pneumoniae and Streptococcus pyogenes. Microorganisms 10:2042. doi:10.3390/microorganisms10102042 36296318 PMC9610097

[49] Watanabe A, Kawada-Matsuo M, Le MN-T, Hisatsune J, Oogai Y, Nakano Y, Nakata M, Miyawaki S, Sugai M, Komatsuzawa H. 2021. Comprehensive analysis of bacteriocins in Streptococcus mutans. Sci Rep 11:12963. doi:10.1038/s41598-021-92370-1 34155274 PMC8217173

[50] Llull D, López R, García E. 2006. Characteristic signatures of the lytA gene provide a basis for rapid and reliable diagnosis of Streptococcus pneumoniae infections. J Clin Microbiol 44:1250–1256. doi:10.1128/JCM.44.4.1250-1256.2006 16597847 PMC1448622

[51] Avery OT, Macleod CM, McCarty M. 1944. Studies on the chemical nature of the substance inducing transformation of Pneumococcal types : induction of transformation by a desoxyribo acid fraction isolated from pneumococcus type iii.. J Exp Med 79:137–158. doi:10.1084/jem.79.2.137 19871359 PMC2135445

[52] Kjos M, Aprianto R, Fernandes VE, Andrew PW, van Strijp JAG, Nijland R, Veening J-W. 2015. Bright fluorescent Streptococcus pneumoniae for live-cell imaging of host-pathogen interactions. J Bacteriol 197:807–818. doi:10.1128/JB.02221-14 25512311 PMC4325099

[53] Maricic N, Dawid S. 2014. Using the overlay assay to qualitatively measure bacterial production of and sensitivity to Pneumococcal bacteriocins. JoVE, no. 91. doi:10.3791/51876-v PMC4235403 25350516

[54] Wick RR, Judd LM, Gorrie CL, Holt KE. 2017. Unicycler: Resolving bacterial genome assemblies from short and long sequencing reads. PLoS Comput Biol 13:e1005595. doi:10.1371/journal.pcbi.1005595 28594827 PMC5481147

[55] Wick RR, Schultz MB, Zobel J, Holt KE. 2015. Bandage: interactive visualization of de novo genome assemblies. Bioinformatics 31:3350–3352. doi:10.1093/bioinformatics/btv383 26099265 PMC4595904

[56] Seemann T. 2014. Prokka: rapid prokaryotic genome annotation. Bioinformatics 30:2068–2069. doi:10.1093/bioinformatics/btu153 24642063

[57] van Heel AJ, de Jong A, Song C, Viel JH, Kok J, Kuipers OP. 2018. BAGEL4: a user-friendly web server to thoroughly mine RiPPs and bacteriocins. Nucleic Acids Res 46:W278–W281. doi:10.1093/nar/gky383 29788290 PMC6030817

[58] Blin K, Shaw S, Steinke K, Villebro R, Ziemert N, Lee SY, Medema MH, Weber T. 2019. antiSMASH 5.0: updates to the secondary metabolite genome mining pipeline. Nucleic Acids Res 47:W81–W87. doi:10.1093/nar/gkz310 31032519 PMC6602434

[59] Bailey TL, Gribskov M. 1998. Combining evidence using p-values: application to sequence homology searches. Bioinformatics 14:48–54. doi:10.1093/bioinformatics/14.1.48 9520501

[60] Krogh A, Larsson B, von Heijne G, Sonnhammer EL. 2001. Predicting transmembrane protein topology with a hidden Markov model: application to complete genomes. J Mol Biol 305:567–580. doi:10.1006/jmbi.2000.4315 11152613

[61] 1994. Fitting a mixture model by expectation maximization, p 28–36. Proc Int Conf Intell Syst Mol Biol. 7584402

[62] Slager J, Aprianto R, Veening JW. 2018. Deep genome annotation of the opportunistic human pathogen Streptococcus pneumoniae D39. Nucleic Acids Res 46:9971–9989. doi:10.1093/nar/gky725 30107613 PMC6212727

[63] Grant CE, Bailey TL, Noble WS. 2011. FIMO: scanning for occurrences of a given motif. Bioinformatics 27:1017–1018. doi:10.1093/bioinformatics/btr064 21330290 PMC3065696

[64] McGinnis S, Madden TL. 2004. BLAST: at the core of a powerful and diverse set of sequence analysis tools. Nucleic Acids Res 32:W20–5. doi:10.1093/nar/gkh435 15215342 PMC441573

[65] Crooks GE, Hon G, Chandonia JM, Brenner SE. 2004. WebLogo: a sequence logo generator. Genome Res 14:1188–1190. doi:10.1101/gr.849004 15173120 PMC419797

[66] Naville M, Ghuillot-Gaudeffroy A, Marchais A, Gautheret D. 2011. ARNold: a web tool for the prediction of Rho-independent transcription terminators. RNA Biol 8:11–13. doi:10.4161/rna.8.1.13346 21282983

[67] Zuker M. 2003. Mfold web server for nucleic acid folding and hybridization prediction. Nucleic Acids Res 31:3406–3415. doi:10.1093/nar/gkg595 12824337 PMC169194

[68] Junges R, Salvadori G, Shekhar S, Åmdal HA, Periselneris JN, Chen T, Brown JS, Petersen FC, Ellermeier CD. 2017. A quorum-sensing system that regulates Streptococcus pneumoniae biofilm formation and surface polysaccharide production. mSphere 2:e00324-17. doi:10.1128/mSphere.00324-17 28932816 PMC5597970

[69] Schmittgen TD, Livak KJ. 2008. Analyzing real-time PCR data by the comparative C(T) method. Nat Protoc 3:1101–1108. doi:10.1038/nprot.2008.73 18546601

[70] Synefiaridou D, Veening JW. 2021. Harnessing CRISPR-Cas9 for genome editing in Streptococcus pneumoniae D39V. Appl Environ Microbiol 87:e02762-20. doi:10.1128/AEM.02762-20 33397704 PMC8105017

[71] Koo BM, Kritikos G, Farelli JD, Todor H, Tong K, Kimsey H, Wapinski I, Galardini M, Cabal A, Peters JM, Hachmann AB, Rudner DZ, Allen KN, Typas A, Gross CA. 2017. Construction and analysis of two genome-scale deletion libraries for Bacillus subtilis. Cell Syst 4:291–305. doi:10.1016/j.cels.2016.12.013 28189581 PMC5400513

[72] Salvadori G, Junges R, Morrison DA, Petersen FC. 2016. Overcoming the barrier of low efficiency during genetic transformation of Streptococcus mitis. Front Microbiol 7:1009. doi:10.3389/fmicb.2016.01009 27458432 PMC4932118

[73] Albert H, Dale EC, Lee E, Ow DW. 1995. Site-specific integration of DNA into wild-type and mutant lox sites placed in the plant genome. Plant J 7:649–659. doi:10.1046/j.1365-313x.1995.7040649.x 7742860

