# Supplementary material for: Inhibition of pneumococcal growth and biofilm formation by human isolates of Streptococcus mitis and Streptococcus oralis
Source: Appl Environ Microbiol. 2025 Feb 26;91(3):e01336-24. doi: 10.1128/aem.01336-24 (PMC11921387; doi:10.1128/aem.01336-24)
Supplement: Supplemental figures — Figures S1 to S10. [file aem.01336-24-s0001.pdf]

Figure S1

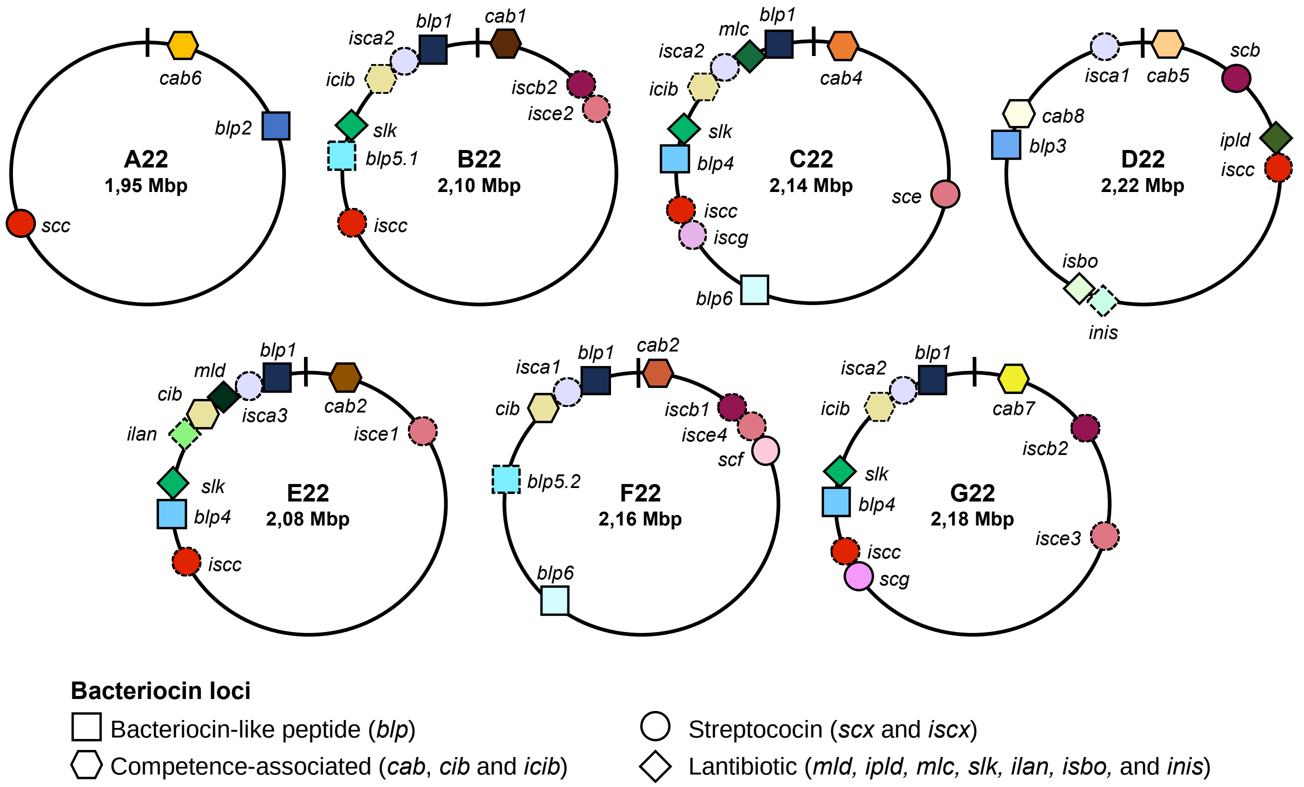

Figure S1. Sixty-one bacteriocin-related loci are differentially distributed across the genomes of strains A22 to G22. Loci lacking bacteriocin genes (but otherwise containing genes characteristic of bacteriocin loci) are represented by dashed lines. Loci of the same type are typically located in similar genomic regions across strains.

Figure S2

A

Streptococcin F locus

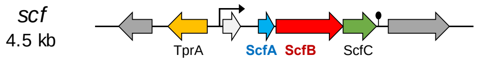


```
                                       20                  40                  60
                                        |                   |                   |
ScaA  M---------------RKLVKIGVASLMVCGILATT--------NALAVWVEGGQ--WNYGVGWT--GTF
ScbA  M--------------KQTVKK-----LALVASIAATLGGSVAVASAAVQYPEGGV--WTYG--SGNGG--
SccA  MLHCCSGCAFFERKNKMKTKKHRLLALALISSF--TLLGA---ASAAVQYPDGGV--WTYG--EGSGGGW
ScdA  M----------------KTSKIILSVLTVIISCSLAC-------PVFADWVSGGN--WSYGGYHNPGNWG
SceA  ML---------------KFRKVVASSLAVL-AFSLAL-------PVLAVSHRGGE--WTYGGHHDPYNWG
ScfA  M--------------KKSIKKIITTSLLTLTLAGAG----GSIVSAATVWYKGTAVYWNYG---RTAGLW
ScgA  M---------------RKLKN-----VLLVSALALSAVTATVSIAETVDV-EGGT--WSYG--YTTGIN-

                   80                  100                 120
                    |                   |                   |
ScaA  GYSDYLHSTRYHTATV---RHGGRTSKDYAKPEAWAIASLTKIPPTGMEYFYGFE--  95
ScbA  AYSNYYHPSKYHSSTVVSR-KTGSSDKGYAGAGGTSRAWIRTSWGEKVAFYYN---V  98
SccA  AFSNYYHGKKYHYSSVVSK-WDSHSDKGSAPAGKTSEAWIWT--------------- 102
ScdA  AFSNYFHDYRWHWSSV-TRASDSKANVGYASAHYTSRSFINTSFGETAYF---NYGF  98
SceA  AFSNYYHGSQYHWAYVGSNERDNQKTV-YAGAHSAAYAFVNTNLGERITF---DAGW  98
ScfA  SYSSVQSSVYEHSATA------NGSFSGWQSPGV--EAHVSKFIGTVGKYL------  92
ScgA  AYSDYYHSYNYHGSRVKNR-NNGDTNTANAESGVWSKAWIWDVWDPATFYYSSNGGY 100
```

B

Streptococcin G locus

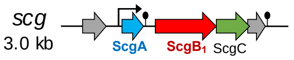

C

Mitilancidin locus

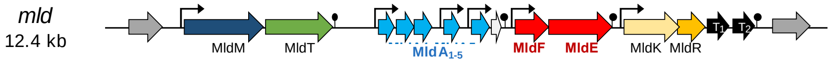


```
                                     20
                                      |
MldA1  GWFTAVQLSL  AGRCGRWFTG  SFECTSNNVS  CGK 33
MldA2  GWFTAVQLSL  AGRCGRWFTG  SFECTSNNVS  CGK 33
MldA3  GWFTAVQLSL  AGRCGRWFTG  SFECTSNHVK  CG- 32
PldA1  GW-TAVQLTL  AGRCGRWFTG  SFECTTNNVK  CG- 31

                                     20
                                      |
MldA4  GGWYSAFKMT  LAGRCGRCFT  CSYECTSNNV  NC 32
PldA3  GWWYTAFKMT  LAGRCGLCFT  CSYECTTNNV  HC 32
```

D

Mitilacticin locus

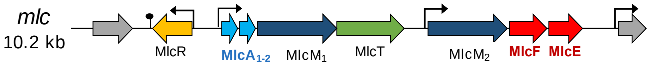


```
                                    20                                  40
                                     |                                   |
MlcA1              MVKKENSYKN  VSWIEEIQDQ  NFNDDVFGAC  STNTFTLSDF  LGNDGGWCTI  SVECMAWCH 59
Lacticin 3147 A1   MNKNEIETQP  VTWLEEVSDQ  NFDEDVFGAC  STNTFSLSDY  WGNNGAWCTL  THECMAWCK 59

                                    20                                  40                                  60
                                     |                                   |                                   |
MlcA2              MM------NN  T--LQLGKYT  EEDMIELTEE  DVNGGAITLV  TPLTPQFVEV  TVAATATICP  TSSCTSRC 60
Lacticin 3147 A2   MKEKNMKKND  TIELQLGKYL  EDDMIELAEG  DESHGG---T  TPATPAISIL  SAYISTNTCP  TTKCTRAC 65
```

(legend on next page)

Figure S2. Organization of gene clusters of novel bacteriocins and alignments with their closest homologues. Novel streptococcin (lactococcin 972-like) loci, named streptococcin F22 (*scf*) and G22 (*scg*), and novel lantibiotic loci, named mitilancidin (*mld*) and mitilacticin (*mlc*), were found. **(A) Streptococcin F (*scf*) locus.** A complete streptococcin locus was found in *S. mitis* strain F22. Noteworthy, the putative bacteriocin had no homology to any streptococcin previously described, which is corroborated by the absence of both bacteriocin and immunity protein homologs from any of the 7,548 pneumococcal genomes analysed **(Table S3)**. The locus encodes *scfA* (bacteriocin), *scfB* (immunity protein), and *scfC* (transporter). A TprA transcriptional regulator and a hypothetical protein are encoded upstream *scf*. Alignment of streptococcin F with known streptococcins (lactococcin 972-like) is shown; **(B) Streptococcin G (*scg*) locus.** A complete streptococcin locus was found in *S. mitis* strain G22. The putative bacteriocin had no homology to any streptococcin previously described, which was corroborated by the absence of both bacteriocin and immunity protein homologs from any of the 7,548 pneumococcal genomes analysed **(Table S3)**. The locus encodes *scgA* (bacteriocin), *scgB* (immunity protein), and *scgC* (transporter). Alignment of streptococcin G with known streptococcins (lactococcin 972-like) is shown in (A); **(C) Mitilancidin (mld) locus.** A gene cluster belonging to class II two-component lantibiotics was found in *S. mitis* strain E22. It encodes a LanM family modification enzyme, MldM, and a peptidase domain-containing ABC transporter, MldT. Downstream of the transporter, there is a tandem array of five open-reading frames, each encoding a putative lantibiotic peptide – $MldA_1$ to $MldA_5$. Of interest, the five predicted peptides are homologous to each other, and the N-terminal leader sequence of each peptide is followed by a GS-, GA-, or AA-motif likely to be the cleavage site [1]. The mature sequences of $MldA_1$ and $MldA_2$ are 100% similar. Downstream of the $mldA_1$-$mldA_5$, there are genes encoding for two putative ABC transporters predicted to be immunity proteins MldF (ATP-binding protein) and MldE (permease), followed by genes encoding for a two-component system MldK (histidine kinase) and MldR (response regulator). The locus resembles the gene cluster encoding pneumolancidin (pld) locus, a functional multipeptide lantibiotic gene cluster previously described in *S. pneumoniae* [1]. The mature peptides of $MldA_1$ to $MldA_3$ and PldA1 share 90% similarity, whereas $MldA_4$ and PldA3 share 84% identity [1]; and **(D) Mitilacticin (*mlc*) locus.** A gene cluster belonging to class II two-component lantibiotics was found in *S. mitis* strain C22. It resembles the gene cluster encoding lacticin 3147 from *L. lactis* [2]. It encodes two distinct peptides, $MlcA_1$ and $MlcA_2$. These peptides are putatively modified by two distinct LanM family enzymes, $MlcM_1$ and $MlcM_2$, encoded downstream the peptide genes and separated by a gene encoding a peptidase domain-containing ABC transporter, MlcT. Downstream the enzymes, there are two genes encoding ABC transporters predicted to be immunity proteins MlcF (ATP-binding protein) and MlcE (permease). Upstream the peptides, there is a gene encoding an XRE-family transcriptional regulator predicted to be MlcR. In contrast with the lacticin 3147 locus from *L. lactis*, the regulator does not form an operon with the immunity proteins as they stand in opposite locations, with *mlcR* being the 5'- and *mlcFE* the 3'-most genes [3]. Peptides $MlcA_1$ and $MlcA_2$ share 64% and 45%, sequence similarity, respectively, to *L. lactis* homologues [3]. Red shading represent non-conserved amino acids between variants.

References

1. Maricic N, Anderson ES, Opipari AE, Yu EA, Dawid S. 2016. Characterization of a multipeptide lantibiotic locus in *Streptococcus pneumoniae*. mBio 7(1): e01656-15.

2. McAuliffe O, Hill C, Ross RP. 2000. Each peptide of the two-component lantibiotic lacticin 3147 requires a separate modification enzyme for activity. Microbiology (Reading) 146 (Pt 9): 2147-54.

3. McAuliffe O, O'Keeffe T, Hill C, Ross RP. 2001. Regulation of immunity to the two-component lantibiotic, lacticin 3147, by the transcriptional repressor LtnR. Mol Microbiol 39(4): 982-93.

Figure S3

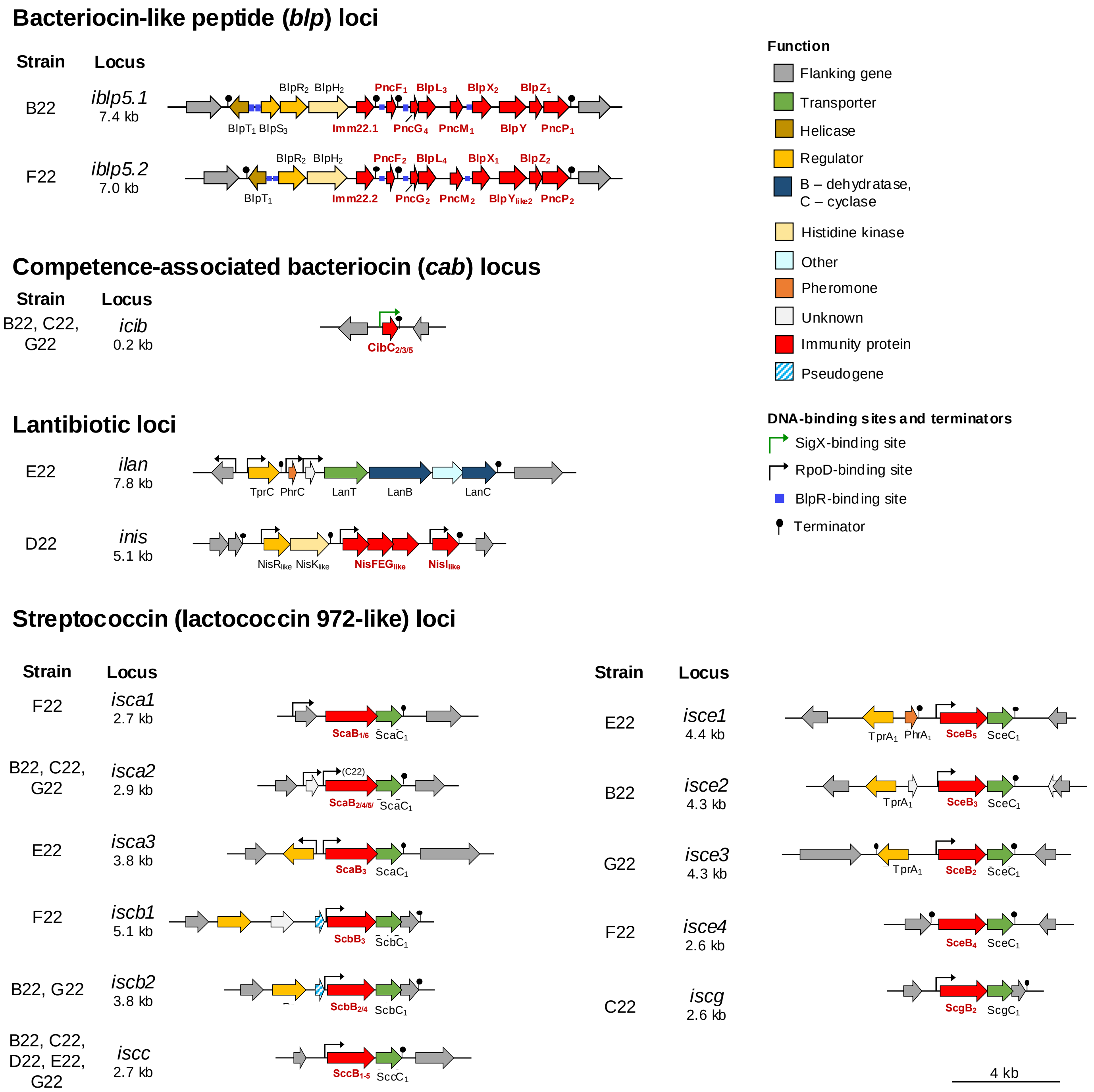

Figure S3. Several incomplete bacteriocin-related loci lacking bacteriocin genes but containing other genes including those encoding for immunity proteins, were found in the genomes of *S. mitis* strains A22 to G22. A schematic representation of the incomplete gene clusters is shown. Genes are colored by function, and flanking genes are represented in grey. Names of putative immunity proteins are indicated in red. Other relevant proteins are indicated in black. Numbers after gene names indicate allelic variants. Locus size (excluding the flanking genes) is indicated next to the locus name. Putative DNA-binding sites (SigX, RpoD, and BlpR) and terminator regions are also represented. Locus tags of the represented regions (genes from left to right) are the following: *iblp5.1* – SMIB22_15810-15680; *iblp5.2* – SMIF22_16160-16040; *icib* – SMIB22_18920-18910, SMIC22_18990-18980, SMIG22_19640-19630; *ilan* – SMIE22_18140-18050; *inis* – SMID22_12350-12270 (immediately downstream locus *isbo*); *isca1* – SMID22_21360-21330, SMIF22_19120-19090; *isca2* – SMIB22_19050-19010, SMIC22_19110-19080, SMIG22_19770-19730; *isca3* – SMIE22_18670-18630; *iscb1* – SMIF22_02720-02780; *iscb2* – SMIB22_02960-03010, SMIG22_02850-02900; *iscc* – SMIB22_14540-14510, SMIC22_14660-14630, SMID22_05300-05330, SMIE22_14010-13980, SMIG22_15400-15370; *isce1* – SMIE22_02080-02040; *isce2* – SMIB22_03330-03280; *isce3* – SMIG22_05940-05980; *isce4* – SMIF22_03080-03050 (immediately downstream locus *scf*); *iscg* – SMIC22_14410-14380.

Figure S4

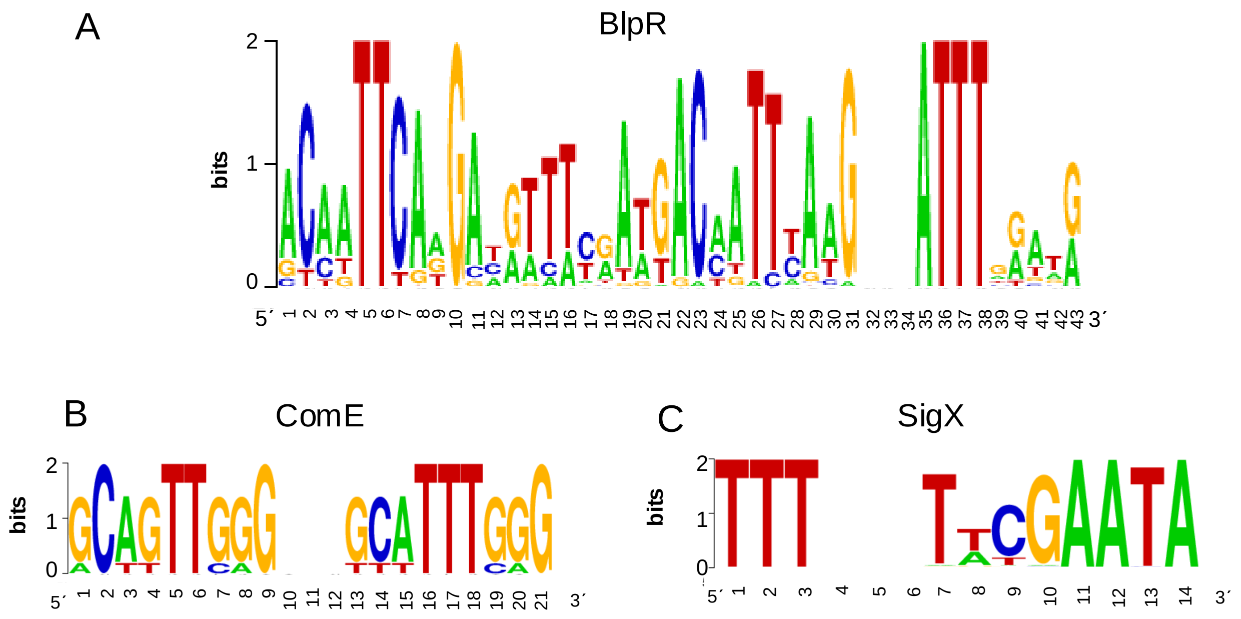

Figure S4. Sequence logos of the binding-sites for BlpR (A), ComE (B) and SigX (C) found in the promoter regions of the bacteriocin-like peptide, and competence-associated loci. Logos were generated with the WebLogo tool developed by Crooks *et al.* (2004), with 58, 7 and 51 binding-sequences for BlpR, ComE and SigX, respectively. In the Y axis the height of the letter shows sequence conservation, while the height of each letter within the stack shows the relative frequency of the corresponding base in that position. Degenerate BlpR-binding sequences (*blpSRH; blpO-like2* and *blpA* operons) were not considered for sequence logo analysis. Blank spaces in the logos represent spacer regions, namely: 0-2 nucleotides for BlpR; 12 nucleotides for ComE and 0-61 nucleotides for SigX binding-sequences.

Figure S5

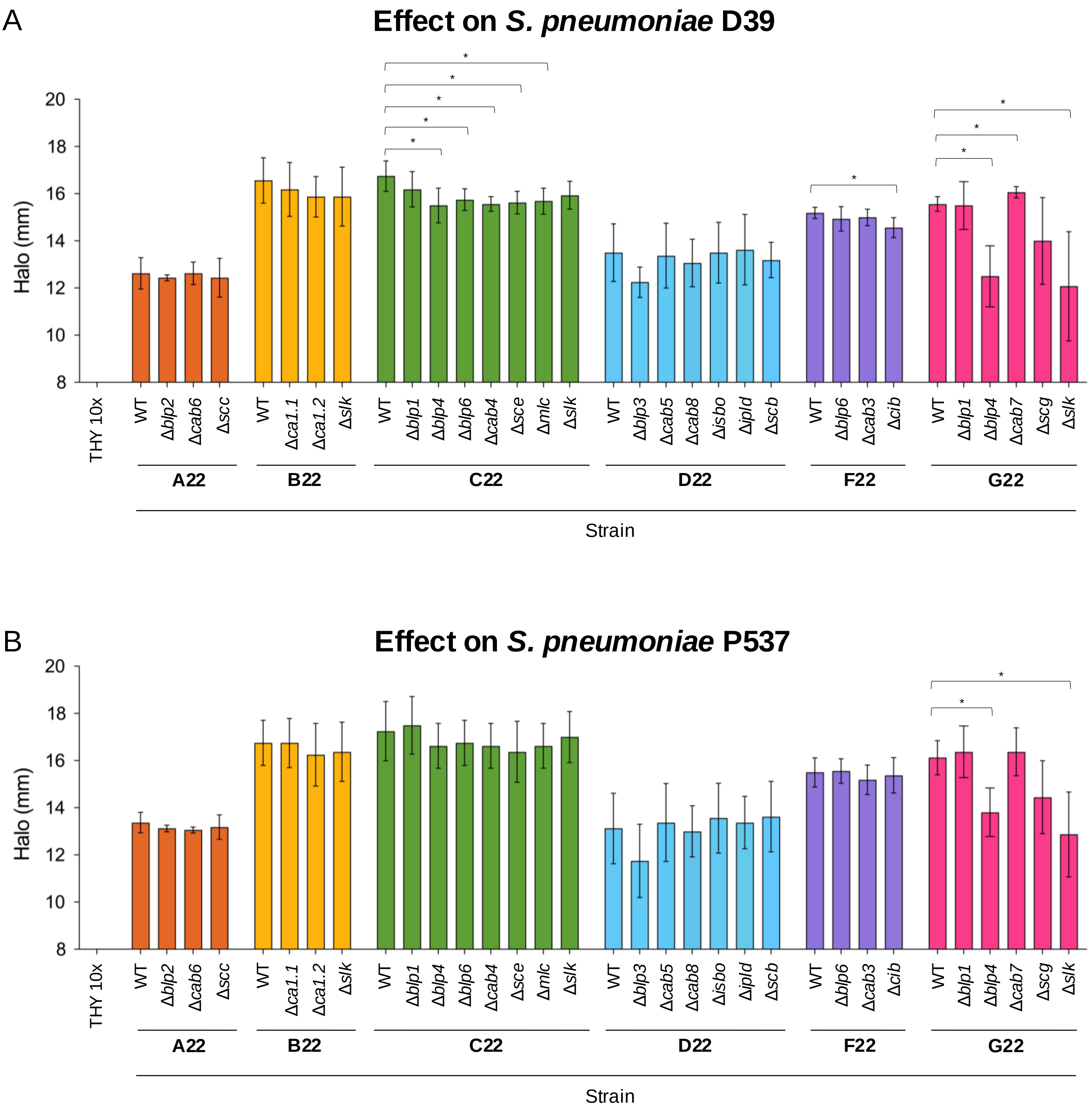

Figure S5. The inhibitory activity of cell-free supernatants (CFS) of *S. oralis* strain A22 and *S. mitis* strains B22, C22, D22, F22, and G22 - wild-type strains and bacteriocin loci deletion mutants - was tested against *S. pneumoniae* D39 and P537 growing in solid media. Well-diffusion assays using 10x CFS or 10x THY (control) against *S. pneumoniae* D39 and P537 were carried out. After overnight incubation with CFS, plates were inspected for inhibition halos. The diameter of the inhibition halos was measured independently by two researchers in four independent experiments. Mean and standard deviation are represented for each condition. The inhibition halo diameter includes the well width of 8mm. *p-value<0.05, **p-value<0.01, Student's t-test.

Figure S6

Effect on *S. pneumoniae* D39

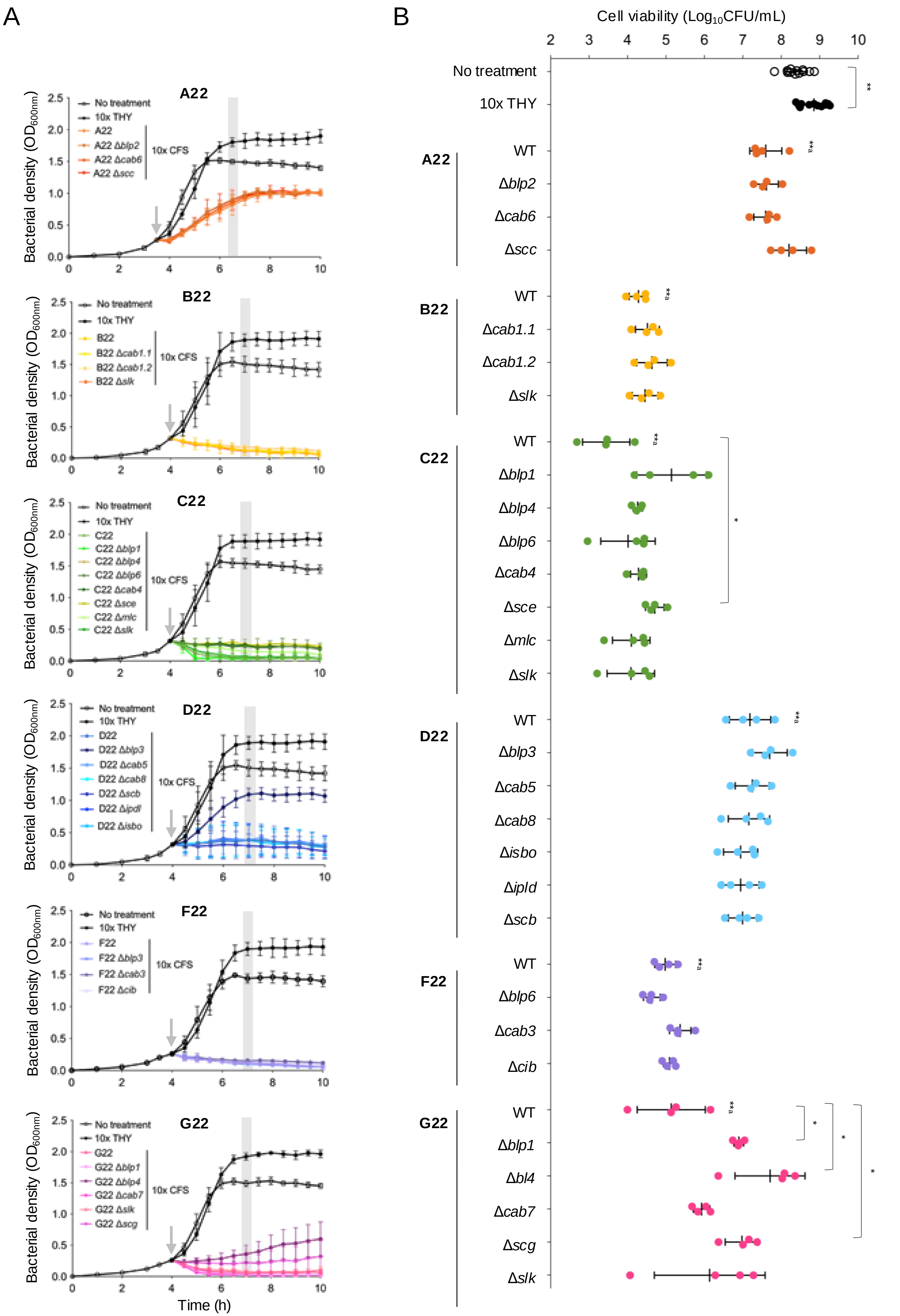

(continues on next page)

Figure S6 Effect on *S. pneumoniae* P537

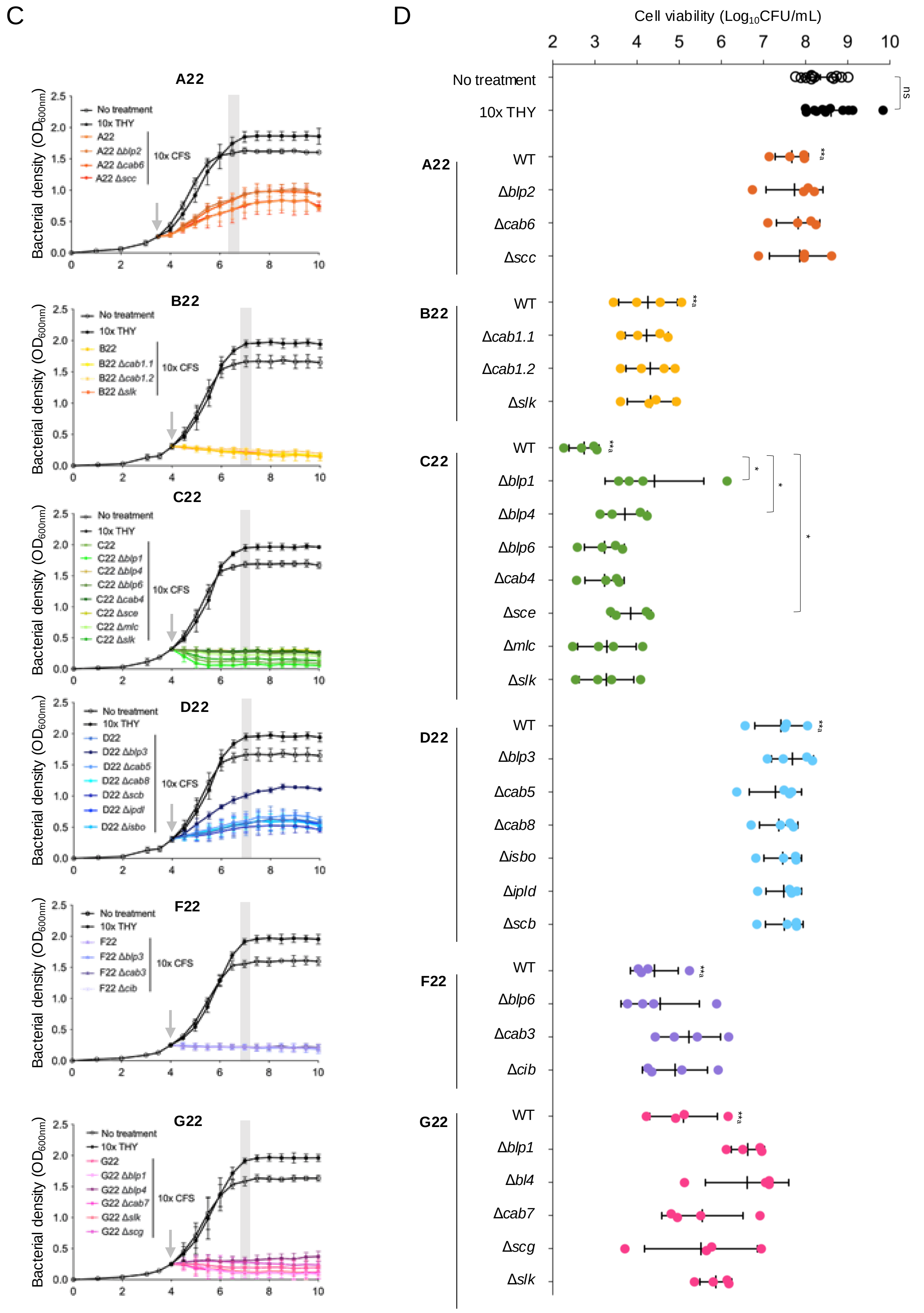

(legend on next page)

Figure S6. The inhibitory activity of cell-free supernatants (CFS) of *S. oralis* strain A22 and *S. mitis* strains B22, C22, D22, F22, and G22 - wild-type strains and bacteriocin loci deletion mutants - was tested against *S. pneumoniae* D39 and P537 growing in liquid media. **(A, C)** The effect of 10x CFS on planktonic growth *S. pneumoniae* D39 and P537 was evaluated. Early exponential phase cultures (OD_{600nm} of 0.3) of D39 and P537 were treated with 10% (v/v) of 10x CFS, 10x THY or remained untreated (as indicated by the grey arrow) and OD_{600nm} was monitored every 30 min. Grey bar indicates the timepoint in which an aliquot was taken for cell viability counts. Mean and standard deviation of at least four biological replicates are represented for each condition. **(B, D)** Cell viability of D39 and P537 was assessed 3 hours post-treatment. Geometric mean and geometric standard deviation of at least four biological replicates are represented for each condition. *p-value<0.05, **p-value<0.01, Mann-Whitney U test with Benjamini and Hochberg correction for FDR; [a] statistical comparison with treatment with 10x THY; ns, not significant.

Figure S7

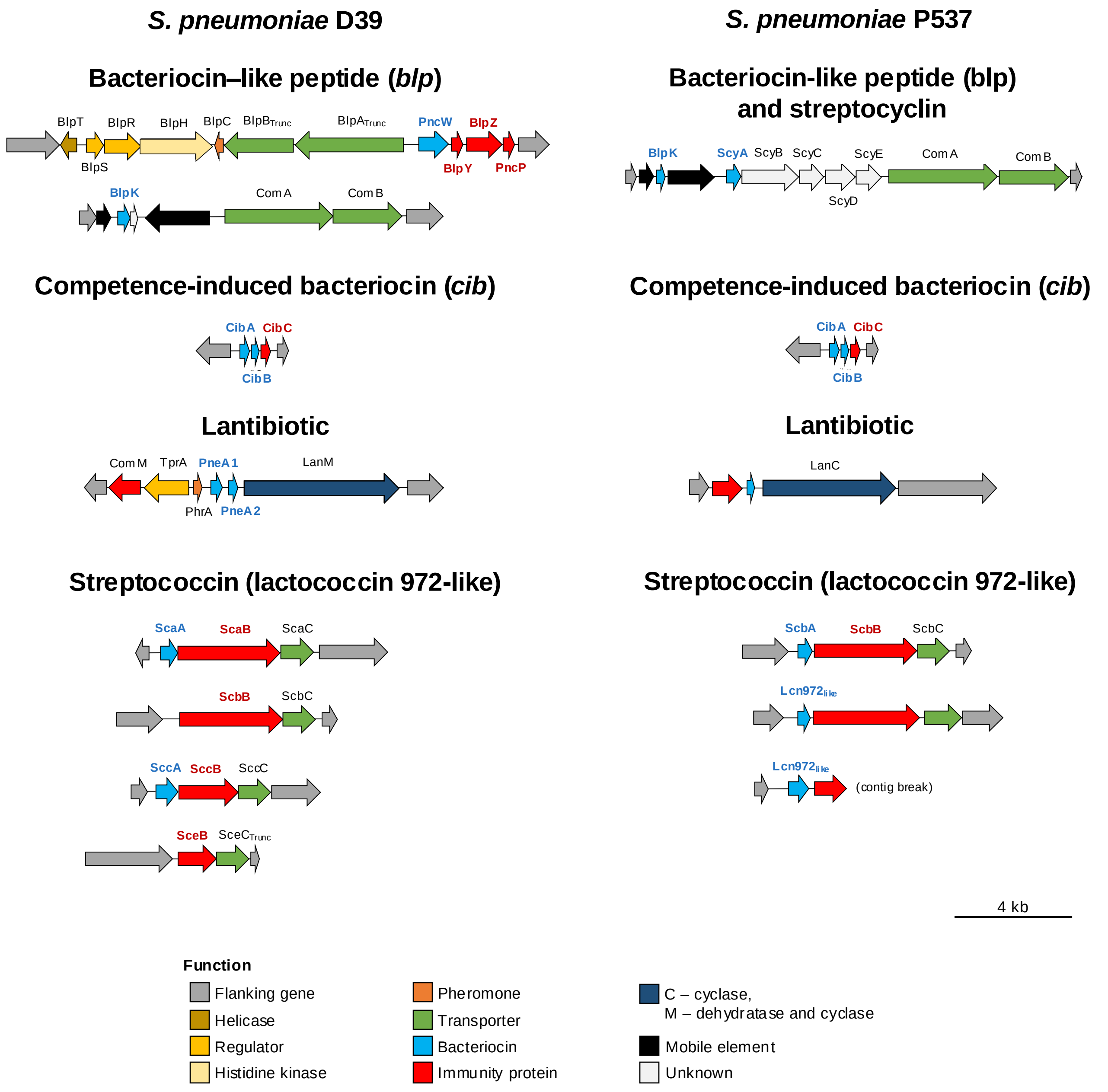

Figure S7. *S. pneumoniae* D39 and P537 genomes encode for a diverse set of bacteriocin-associated loci. Five types of loci were found: bacteriocin-like peptide *(blp),* competence-induced bacteriocin (*cib*), lantibiotic, streptococcin (lactococcin 972-like) and streptocyclin. *S. pneumoniae* P537 lacks *blp* due to a whole-locus deletion. Genes are colored by function. ‘ – truncated gene.

Figure S8

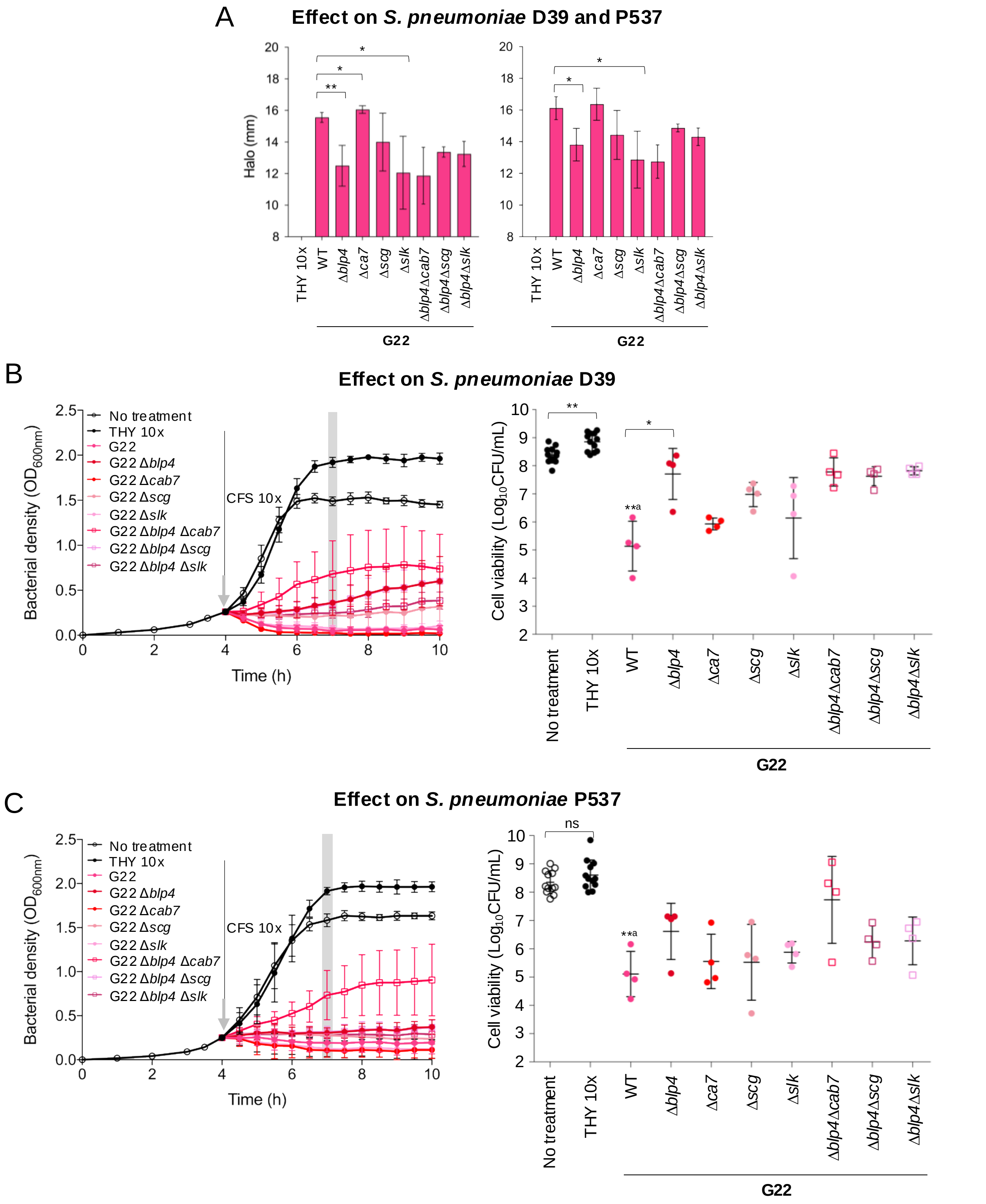

Figure S8. The inhibitory activity of strain G22 is driven by an additive activity of several loci. (A) Well-diffusion assay using CFS 10x or THY 10x (control) against *S. pneumoniae* D39 and P537. After overnight incubation with CFS, plates were inspected for inhibition halos. The diameter of the inhibition halos was measured independently by two researchers in four independent experiments. Mean and standard deviation are represented for each condition. The inhibition halo diameter includes the well width of 8mm. *p-value<0.05, **p-value<0.01, Student's t-test with Benjamini and Hochberg correction for FDR. **(B and C)** The effect of CFS 10x on planktonic growth *S. pneumoniae* D39 **(B)** and P537 **(C)** was assessed. Early exponential phase cultures (OD_{600nm} of 0.3) of D39 and P537 were treated with CFS 10x, THY 10x or remained untreated (as indicated by the grey arrow) and OD_{600nm} was monitored every 30 min (left and middle panels). Grey bar indicates the timepoint in which an aliquot was taken for cell viability counts. Mean and standard deviation of at least four biological replicates are represented for each condition. Cell viability of D39 and P537 was assessed 3 hours post-treatment (right panel). *p-value<0.05, **p-value<0.01, Mann-Whitney U test with Benjamini and Hochberg correction for FDR; [a] statistical comparison with treatment with 10x THY; ns, not significant.

Figure S9

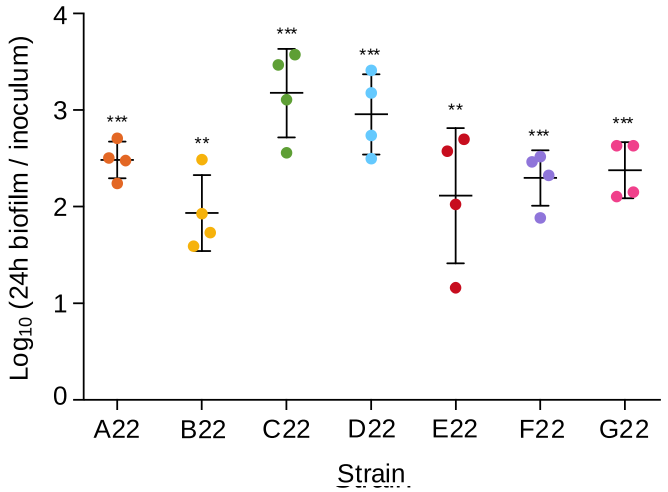

Figure S9. *S. oralis* strain A22 and *S. mitis* strains B22 to G22 grow in biofilms on an abiotic surface. Strains A22-G22 were inoculated on 24-well plates at 10^5 CFU/mL in 2.5 mL of THY supplemented with catalase (1600 U/mL). Plates were incubated for 24h at 34°C in 5% CO_2. On the following day, supernatants were carefully removed, and biofilms were resuspended in 500 µL of PBS. Serial dilutions were plated on blood-agar plates and incubated for 24h at 37°C in 5% CO_2 for biofilm viability (CFU counts) assessment. Colony forming units were counted after overnight incubation. The ratio between the bacterial density (CFU/mL) observed for each strain after a 24h biofilm and the inoculum is shown. Four independent experiments were performed. Statistical analysis was done between the bacterial density of the inoculum and of the 24h biofilm for each strain. *p<0.05, **p<0.01, ***p<0.001, ratio paired t test; ns, not significant.

Figure S10

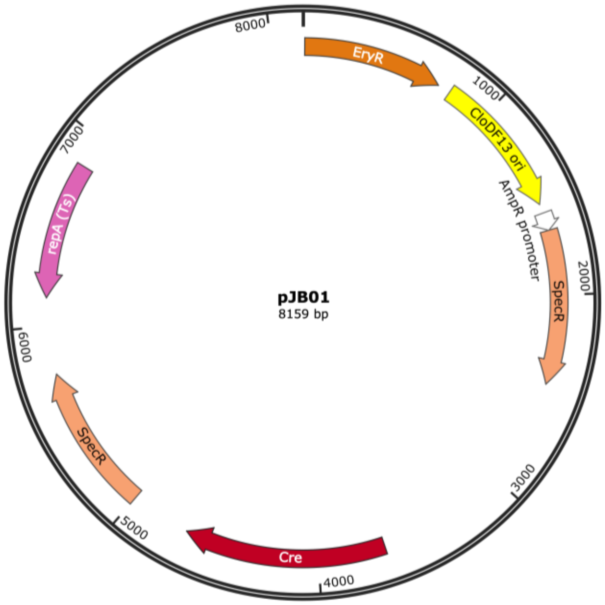

Figure S10. Map of plasmid pJB01. The plasmid contains two replication origins, one for *E. coli* and another for Gram-positive bacteria. In Gram-positive bacteria, the plasmid replicates at 30°C and is cured at 37°C or above. pJB01 has two selection markers for streptococci (spectinomycin and erythromycin) and one for *E. coli* (spectinomycin). The plasmid constitutively encodes *cre.*
